# Using bacterial pan-genome-based feature selection approach to improve the prediction of minimum inhibitory concentration (MIC)

**DOI:** 10.3389/fgene.2023.1054032

**Published:** 2023-05-30

**Authors:** Ming-Ren Yang, Shun-Feng Su, Yu-Wei Wu

**Affiliations:** ^1^ Graduate Institute of Biomedical Informatics, College of Medical Science and Technology, Taipei Medical University, Taipei, Taiwan; ^2^ Department of Electrical Engineering, National Taiwan University of Science and Technology, Taipei, Taiwan; ^3^ Clinical Big Data Research Center, Taipei Medical University Hospital, Taipei, Taiwan; ^4^ TMU Research Center for Digestive Medicine, Taipei Medical University, Taipei, Taiwan

**Keywords:** feature selection, pan-genome, antimicrobial resistance (AMR), minimum inhibitory concentration, MIC, *Salmonella enterica*, regression

## Abstract

**Background:** Predicting the resistance profiles of antimicrobial resistance (AMR) pathogens is becoming more and more important in treating infectious diseases. Various attempts have been made to build machine learning models to classify resistant or susceptible pathogens based on either known antimicrobial resistance genes or the entire gene set. However, the phenotypic annotations are translated from minimum inhibitory concentration (MIC), which is the lowest concentration of antibiotic drugs in inhibiting certain pathogenic strains. Since the MIC breakpoints that classify a strain to be resistant or susceptible to specific antibiotic drug may be revised by governing institutes, we refrained from translating these MIC values into the categories “susceptible” or “resistant” but instead attempted to predict the MIC values using machine learning approaches.

**Results:** By applying a machine learning feature selection approach on a *Salmonella enterica* pan-genome, in which the protein sequences were clustered to identify highly similar gene families, we showed that the selected features (genes) performed better than known AMR genes, and that models built on the selected genes achieved very accurate MIC prediction. Functional analysis revealed that about half of the selected genes were annotated as hypothetical proteins (i.e., with unknown functional roles), and that only a small portion of known AMR genes were among the selected genes, indicating that applying feature selection on the entire gene set has the potential of uncovering novel genes that may be associated with and may contribute to pathogenic antimicrobial resistances.

**Conclusion:** The application of the pan-genome-based machine learning approach was indeed capable of predicting MIC values with very high accuracy. The feature selection process may also identify novel AMR genes for inferring bacterial antimicrobial resistance phenotypes.

## Introduction

Antimicrobial resistance (AMR) of bacterial pathogens is a global issue associated with high morbidity and mortality (Akova, 2016; Frieri et al., 2017). According to a 2013 US Center for Disease Control and Prevention (CDC) report, at least 23,000 people died due to antibiotic-resistant infections (Prestinaci et al., 2015). The same report also estimated that treating AMR-related infectious diseases may cost as much as 55 billion USD per year, indicating the huge losses caused by AMR pathogens.

There are multiple factors related to the rise of the AMR pathogens. Drug resistances naturally occur when microorganisms evolve mechanisms to protect themselves from antimicrobial agents. This is especially the case during the treatment phase of infectious diseases, in which pathogens may quickly develop resistance against the antibiotic drugs and impede the entire medical process. Very often patients with infectious diseases are unnecessarily prescribed broad spectrum antibiotics due to the inability of predicting the antimicrobial resistance patterns as quickly as possible for the pathogens (Akova, 2016). This practice may lead to a dramatic increase of bacterial resistance to administered drugs, and, if not controlled, may result in the spread of the drug-resistant bacteria to other patients and the environments, eventually rendering the antibiotic drugs less useful (Akova, 2016).

Antibiotic resistance in microbial pathogens is largely related to their genetic content (Botelho and Schulenburg, 2021), and one of the most common approaches for identifying potential AMR pathogens is through the annotation of drug resistance genes by comparing genes against databases to infer genotypic resistance profiles of the pathogens (Peterson and Kaur, 2018). However, the major limitation of the database-based approach is that novel resistance genes and mechanisms cannot be uncovered using database-based methods (Berman and Riley, 2013).

Pan-genome-based AMR data mining approaches are becoming more and more popular in recent years. The concept of pan-genome analysis is the appropriate approximation of genetic elements to describe a species (Medini et al., 2005). In a nutshell a pan-genome is a collection of all genes found in a bacterial species. Upon considering multiple strains in the same species, the genes can then be classified into core genes (i.e., present in all or most of the strains) and accessory genes (or auxiliary genes, in which genes can only be identified in a portion of the bacterial genomes) based on the gene presence/absence patterns (Medini et al., 2005; McInerney et al., 2017). Due to its nature in representing multiple strains for a bacterial species, pan-genomes are very commonly applied to conduct phenotypic association studies such as AMR analysis. For example, Scoary is an analysis software that scores pan-genome components based on their associated phenotypes (Brynildsrud et al., 2016). PARMAP is another pan-genome-based computational framework for predicting antimicrobial resistance (Li et al., 2020). These and other tools (Lees et al., 2018; McCarthy and Fitzpatrick, 2019; Tonkin-Hill et al., 2020) show that pan-genome analysis can be very useful in drawing antimicrobial resistance genotype/phenotype associations.

Since pan-genome data is, by its nature, a collection of gene presence/absence patterns of different strains within a species, it can be converted into machine learning-applicable format very easily. Machine learning approaches can then be applied on the pan-genome data to build prediction models for phenotypes such as drug resistance profiles of pathogens based on the entire collection of gene set. The main difference between the traditional approach (i.e., mining and analyzing known resistance genes) and the pan-genome-based approach is that the pan-genome is capable of incorporating and analyzing all possible genes instead of just known resistance genes, thereby avoiding the “uncharacterized resistance genes” problem. Attempts have been made to predict antimicrobial resistances based on the constructed pan-genomes and yielded good prediction results (Kavvas et al., 2018; Moradigaravand et al., 2018; Maguire et al., 2019; Hyun et al., 2020; Khaledi et al., 2020). We have also developed pan-genome-based approaches that predicted antimicrobial resistance profiles for different pathogens using machine learning feature selection methods (Her and Wu, 2018; Yang and Wu, 2022; Yang and Wu, 2023). These examples demonstrated that applying machine learning approaches on the pan-genome data may enhance the prediction of AMR pathogens by including both known and uncharacterized AMR genes.

Assessment of bacterial susceptibility to antimicrobials is based on either inhibition zones or MIC values. By definition the MIC values are the lowest concentrations of the water-soluble antibiotic drug to inhibit the growth of specific microbial strains (Mann and Markham, 1998). Upon obtaining the MICs, the values can then be interpreted by breakpoints (which are source-specific such as CLSI veterinary medicine standards) for specific pathogens established by organizations such as the Clinical and Laboratory Standards Institutes (CLSI), the European Committee on Antimicrobial Susceptibility Testing (EUCAST), or other institutes like the U.S.-centric CDER (U.S. Food and Drug Administration Center for Drug Evaluation and Research) (Humphries et al., 2019). In other words, the “resistant” and “susceptible” phenotypes were translated from the defined breakpoints.

One major drawback for such MIC-phenotype translation is that breakpoints may be revised based on gathered clinical data, pharmacokinetic-pharmacodynamic property, or MIC distributions (Hombach et al., 2012). For example, the CLSI has revised breakpoints for several anaerobic bacteria in 2010/2011, including the zone diameter breakpoints for Enterobacteriaceae, *Pseudomonas aeruginosa*, and *Acinetobacter baumannii* against third-generation cephalosporins, carbapenems, and fluoroqinolones (Hombach et al., 2012). In 2019 the CLSI again revised ciprofloxacin and levofloxacin breakpoints for Enterobacteriaceae and *P. aeruginosa*, daptomycin breakpoint for *Enterococcus* spp., and ceftaroline breakpoint for *Staphylococcus aureus* (Humphries et al., 2019). These revisions showed that the “resistant” or “susceptible” phenotypes may be outdated and therefore need to be consistently updated with the publication of new guidelines. In case that machine learning models would predict categories (susceptible/resistant) instead of MIC-valus, the models that were trained to outdated breakpoints might predict wrong phenotypes and may need to be re-trained with the updated phenotype annotations.

Several attempts have been made to conduct such regression tasks. For example, Nguyen et al. has extracted k-mers and applied XGBoost regression model to predict MIC values (Nguyen et al., 2018), and Pataki et al. has extracted known resistance genes and used random forest and linear regression models to conduct feature selection and MIC regression (Pataki et al., 2020). However Nguyen et al. only considered ±1 two-fold dilution factor, and Pataki et al. only incorporated known resistance genes without considering the entire genome content. To the best of our knowledge a more universal approach that predicts a wider range of MIC values based on the entire genome content is still needed to take more diverse pathogens with a wider range of MIC distribution into account.

In this work we attempted to predict the MIC values instead of resistant/susceptible categories based on a pan-genome-based machine learning feature selection algorithm in order to avoid the problem of potentially-outdated resistant/susceptible categories. Since MIC values were numerically distributed, the model was designed to regress the values based on the pan-genome content. We applied the model on a *Salmonella enterica* dataset as a test example since *S. enterica* is a zoonotic pathogen that may colonize animals, humans, and plants and may also be found in the environments (Knodler and Elfenbein, 2019). Approximately 1.4 million cases of salmonellosis occur on human in the United States each year (Brenner et al., 2000) and caused 155,000 deaths annually (Eng et al., 2015). The drug-resistant *Salmonella* was also associated with many outbreaks in the United States (Nair et al., 2018). We therefore hope to develop a computational methodology that allows accurate detection of pathogen resistance profiles, which may be able to control the outbreaks in time. In this manuscript we showed that the protein-based pan-genome machine learning model was capable of predicting MIC values with very good accuracy, and that the feature selection approach was able to extract highly-associated genes for downstream analysis.

## Materials and methods

### Genome collection and annotation

Fasta files of both genomes (.fna files) and translated proteins (.faa files) of *S. enterica* strains were downloaded from the PATRIC database (Wattam et al., 2017), which was one of the most comprehensive antibiotic resistance databases that consisted of both genome data and drug resistance metadata. We made sure that only data entries consisted of the term “*Salmonella enterica*” in its species name were downloaded such that no other *Salmonella* species were included in this study. The qualities of the genomes were checked by 1) checkM v1.1.3 (Parks et al., 2015), and 2) mapping 16S ribosomal RNA gene obtained from the *S. enterica* NCBI reference genome (*S. enterica* subsp. *enterica* serovar Typhimurium str. LT2; NCBI acc. NC_003197.2) against the genomes using BLASTN (Altschul et al., 1997). Only genomes with checkM completeness > 95%, contamination < 5%, and 16S rRNA BLAST identity > 99% were considered in the ongoing analysis. The complete genome ID list and accompanying metadata (including their corresponding NCBI genome accession IDs), which was compiled from the metadata file “genome_metadata” downloaded from the PATRIC ftp site, is available in Supplementary Table S1.

The protein fasta files (.faa) of the *S. enterica* strains that passed the genome quality checks were collected and clustered at 95% identity using CD-HIT v4.8.1 software (Li and Godzik, 2006) (parameter: -c 0.95 -d 0 -M 16000) in order to generate the *S. enterica* pan-genomes. All genes located on plasmids as well as on the chromosomes were included in the analysis (will be discussed in Discussion). The amount of core and accessory genes were then estimated, in which core genes were defined as those that appear in all (100%) strains while accessory genes were those that appeared only in some but not all strains. The gene accumulation curve distributions of the pan-, core-, and accessory-genes were estimated by randomly sampling genomes and cumulatively calculating the number of genes. The sampling process was repeated ten times to obtain an averaged number of genes for the distributions. The known AMR genes were identified by annotating known AMR genes using both CARD/RGI v5.2.0 (Alcock et al., 2019) and Resfams v1.2 (Gibson et al., 2015) on the representative sequences of the CD-HIT results; genes that were discovered by either CARD/RGI or Resfams were considered as known AMR genes.

The drug resistance/susceptibility metadata (PATRIC_genomes_AMR.txt) that included the minimum inhibitory concentration (MIC) of *S. enterica* strains toward each drug was also downloaded from the ftp site of the PATRIC database (ftp://ftp.bvbrc.org/), in which strains with MIC information were obtained regardless of the lab typing method for MIC (will be discussed in Discussion). Since the MIC values were measured by gradual dilution of the drug concentrations, the values were usually distributed in the power of 2 (say, 0.25, 0.5, 1, 2, 4, 8, 16, 32, 64, etc.). As a result, the downloaded MIC values were adjusted by log2 when the values were associated with the genome strains. Special care was taken to deal with signs other than “=” or “<“==”.>==”. For greater than sign (>), the log2 adjusted MIC values were incremented by 1. Similarly, for the less than sign (<), the log2-adjusted MIC values were subtracted by 1. On the other hand the greater than or equals to sign (≥) and less than or equals to sign (≤) remained unchanged following (Nguyen et al., 2018). For example, for the value “>256,” which means that the MIC breakpoint was above 256, the log2-adjusted MIC values for this entry is adjusted to be $\left(\log_{2}256\right)+1=8+1=9$ . General rules for adjusting MIC values by log2 was shown in the following equation, where *x* represented the *de facto* MIC value.

$$Transformed\;MIC=\left\{\begin{array}{cc} \left(\log_{2}x\right)+1, & ifMICannotatedas>x\\ \log_{2}x, & ifMICannotatedas=x,==x,\leq x\;or\geq x\\ \left(\log_{2}x\right)-1, & ifMICannotatedas\;<x \end{array}\right.$$

### Machine learning feature selection approach for MIC prediction

Since the gene clustering tool, CD-HIT, is capable of putting highly similar genes (95% amino acid identity) into the same group, each group can be viewed as a gene “cluster” that encompasses genes that are highly similar to each other at sequence level. One can think of the gene clusters as potential gene families since the genes within a cluster are very similar and may be orthologous proteins with identical or very similar functions. On the other hand clustering algorithm may partition sequences into different biologically meaningful groups, allowing the function prediction of genes (Demuth et al., 2006).

To perform machine learning tasks for predicting MICs, the gene clusters were integrated with the log2- adjusted MIC values to build machine learning tables. The tables were constructed as follows. For each table, columns represented the gene clusters (e.g., Cluster1, Cluster2, etc.) while rows indicated different *S. enterica* strains. The presence/absence pattern of the gene clusters were then extracted from the gene clustering results, in which 1 meant presence of the gene cluster for corresponding strains and 0 otherwise. The log2-adjusted MIC values of the strains for each drug were specified as an extra column. Strains without MIC annotations were removed from each of the drug tables; gene clusters that cannot be associated with any strains after removing strains without MIC annotations were also purged from the tables. Since the MIC value distributions for each drug were different, distinct tables were created for different drugs. Only drugs that were associated with at least 1,000 strains with MIC entries were included into the ongoing analysis, including: amoxicillin/clavulanic acid, ampicillin, azithromycin, cefoxitin, ceftiofur, ceftriaxone, chloramphenicol, ciprofloxacin, gentamicin, kanamycin, nalidixic acid, streptomycin, sulfisoxazole, tetracycline, and trimethoprim/sulfamethoxazole. See Supplementary Table S2 for detailed numbers of strains of the drug datasets.

Feature selection was described as the process of obtaining and selecting relevant features from the original feature set for making better predictions in the dataset (Cai et al., 2018). We utilized the idea of feature selection to find relevant gene clusters for predicting MIC values. eXtreme Gradient Boosting (XGBoost), a scalable tree-based machine learning algorithm that combines the advantages of both Bagging and Boosting (Chen and Guestrin, 2016), was used for feature selection purpose. Specifically, the function “XGBRegressor” included in the Python XGBoost package was called to fit the log2-adjusted MIC values by the gene presence/absence patterns with default parameters (n_estimators = 100, max_depth = 6).

After applying the XGBoost feature selection approach, all features with gini-importance > 0 were extracted and sorted by descending importance order, which were then extracted, one-by-one cumulatively, to feed into a Random Forest regression model (scikit-learn package (Pedregosa et al., 2011); n_estimators = 100, max_depth = None) in order to find the feature set that yielded the best regression performances. A 10-fold cross validation approach was utilized to obtain the regression outcome, and the predicted MIC values were yielded by averaging the results of all 10-fold models. The goodness-of-fit of the regression models were evaluated by calculating the R-squared value [*R* ^2^; indicating the proportion of variance in the dependent variable explained by the model (Kasuya, 2019)] and root-mean-square error (RMSE) and conducted Pearson correlation analysis on the pairs of predicted and actual MIC values. The best feature sets for the drugs were selected as the one that maximized the *R* ^2^ values. The codes for running feature selection and regression on the datasets is available at https://github.com/mingren0130/regression. The gene clustering CD-HIT files for generating the pan-genome and the gene presence/absence tables are available at https://doi.org/10.6084/m9.figshare.21913689.v1.

## Results

**FIGURE 1:**
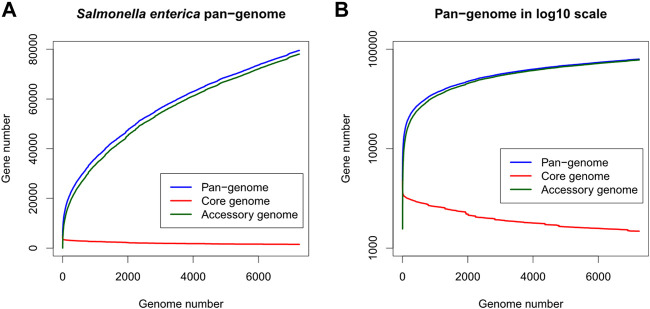
Gene number distribution of the *Salmonella enterica* pan-genome, in which **(A)** shows the number of genes in the *y*-axis while **(B)** shows *y*-axis as log_10_ scale. The *x*-axis indicates the number of genomes. Blue, red, and green colors represent the distributions of pan-, core-, and accessory-genes respectively.

Totally 7,712 *S. enterica* genomes were downloaded from the PATRIC database. After removing 463 low-quality and potentially contaminated genomes, protein-coding genes in the remaining 7,249 genomes were clustered into 79,536 gene clusters, of which 1,479 and 78,057 clusters belonged to the core and accessory genes respectively. The *S. enterica* pan-genome plot shown in Figure 1 demonstrated that the cumulative counts of accessory genes kept growing with the number of genomes while the core gene number remained almost stationary (the minimum, 1st quantile, median, mean, and 3rd quantile numbers of core genes are 1,479, 1,609, 1831, 1961, and 2,329 respectively, indicating that the number of core genes changed very slightly compared to the entire pan-genome or the accessory genes, especially after adding just one-fourth of genomes into the pan-genome). The rapid growth of accessory genes indicated that the gene pool of *S. enterica* was likely unlimited and constituted the definition of open-pan-genome (Costa et al., 2020).

**FIGURE 2:**
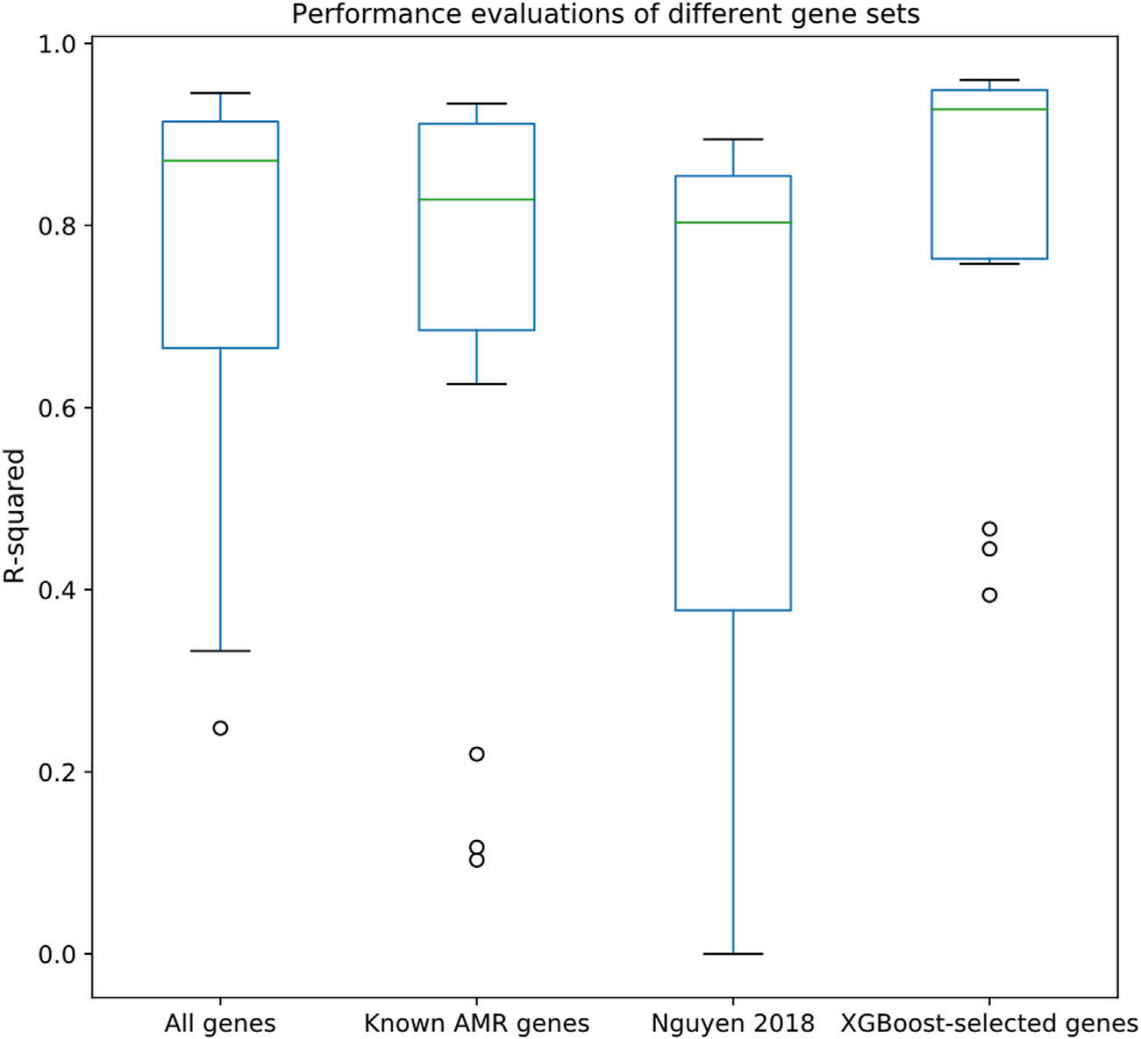
Performance evaluations of different gene sets, including all genes, known AMR genes, the k-mer-based regression approach proposed by (Nguyen et al., 2018), and XGBoost-selected genes. *Y*-axis indicates *R* ^2^ evaluated by Random Forest regression through 10-fold cross validation.

After associating the drug MIC data with the strains in the pan-genome, we selected 15 drugs that satisfied our selection criteria (>1,000 MIC entries; see Methods section). An XGBoost feature selection approach was conducted on the constructed pan-genome gene presence/absence tables (see Methods) to identify meaningful features; the features were then extracted to perform regression tasks using Random Forest regression approach. As shown in Figure 2, regression made on XGBoost-selected genes performed better than using all genes, known AMR genes, or a k-mer-based regression approach (Nguyen et al., 2018). The *R* ^2^ metric, which can be used to evaluate the goodness-of-fit of the prediction outcome, clearly demonstrated that XGBoost-selected genes (*R* ^2^ median = 0.93, 1st quantile = 0.77, and 3rd quantile = 0.95) performed better than all genes (*R* ^2^ median = 0.85, 1st quantile = 0.69, and 3rd quantile = 0.75), known AMR genes (*R* ^2^ median = 0.83, 1st quantile = 0.69, and 3rd quantile = 0.91), and the k-mer-based approach (*R* ^2^ median = 0.80, 1st quantile = 0.38, and 3rd quantile = 0.86). Similar trends can also be observed in the Pearson correlation analysis and RMSE statistics (Supplementary Figures S1, S2), in which the XGBoost-selected genes achieved highest Pearson correlation coefficient and lowest RMSE for most of the drug datasets. Detailed evaluation results are available in Supplementary Tables S3–S5.

**FIGURE 3:**
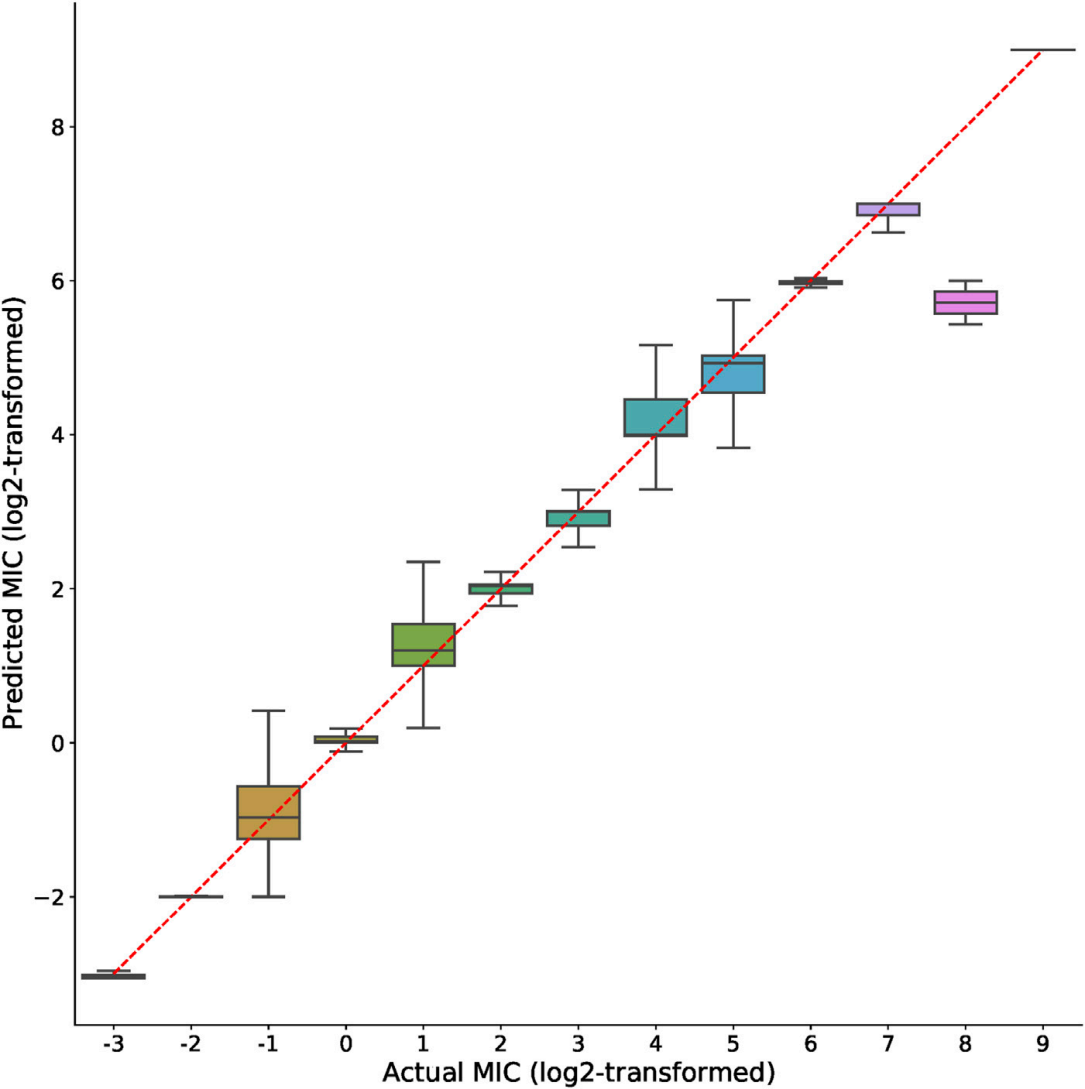
Comparison of actual and predicted MIC values. Boxplots represent the prediction distributions of a distinct MIC. The ticks shown in *X* and *Y*-axis indicate log_2_-transformed MIC values. For better visualization the plot only includes integer entries after the log_2_-transformation of MIC values.

The actual and predicted MIC values using XGBoost-selected genes were also compared side-by-side. As shown in the boxplots in Figure 3, the predicted MIC values were highly correlated with the actual MIC values, indicating the efficacy of the selected features in accurately predicting MICs across very wide value ranges (from 2^−3^–2^9^). This result along with the high *R* ^2^ values (Figure 2) clearly supported the notion that the XGBoost-selected genes may serve as good MIC predictors.

**FIGURE 4:**
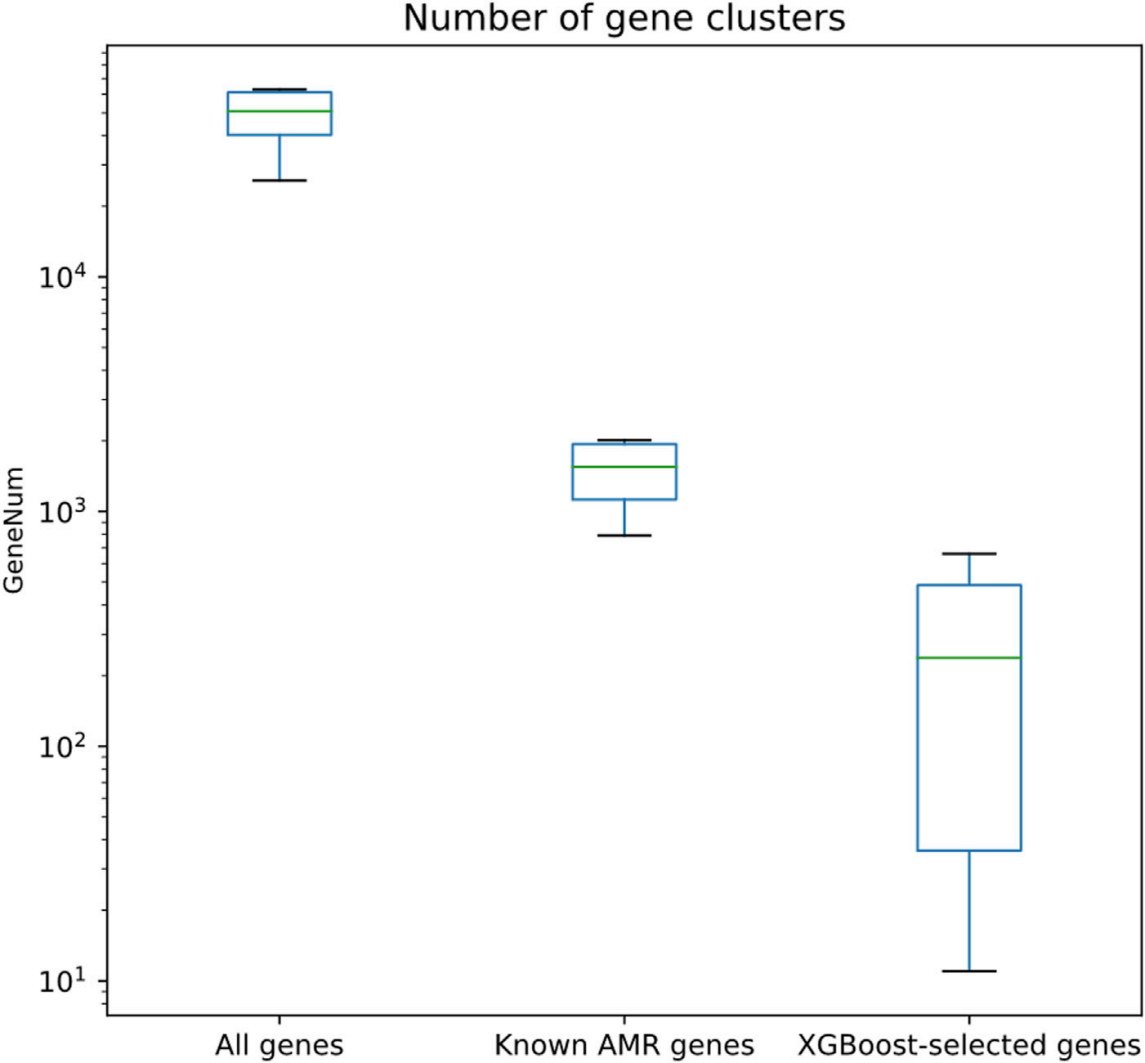
Quantitative assessment of the numbers of gene clusters for different gene sets, including all genes, known AMR genes, and genes selected by XGBoost algorithm.

Besides better regression performances, the numbers of XGBoost-selected genes were clearly fewer than all other gene sets. As shown in Figure 4 and Supplementary Table S6, the numbers of XGBoost-selected genes were significantly lower than all gene set and known AMR gene set (Wilcoxon rank-sum test *p* << 0.001 for both comparisons). The significantly lowered number of genes (1st quantile, median, and 3rd quantile of the gene number distribution are 36, 238, and 486; the minimum and maximum number of genes are 11 and 660) indicated that the XGBoost algorithm may be able to find smaller set of genes that were able to predict more precise MIC values (Figure 2).

**FIGURE 5:**
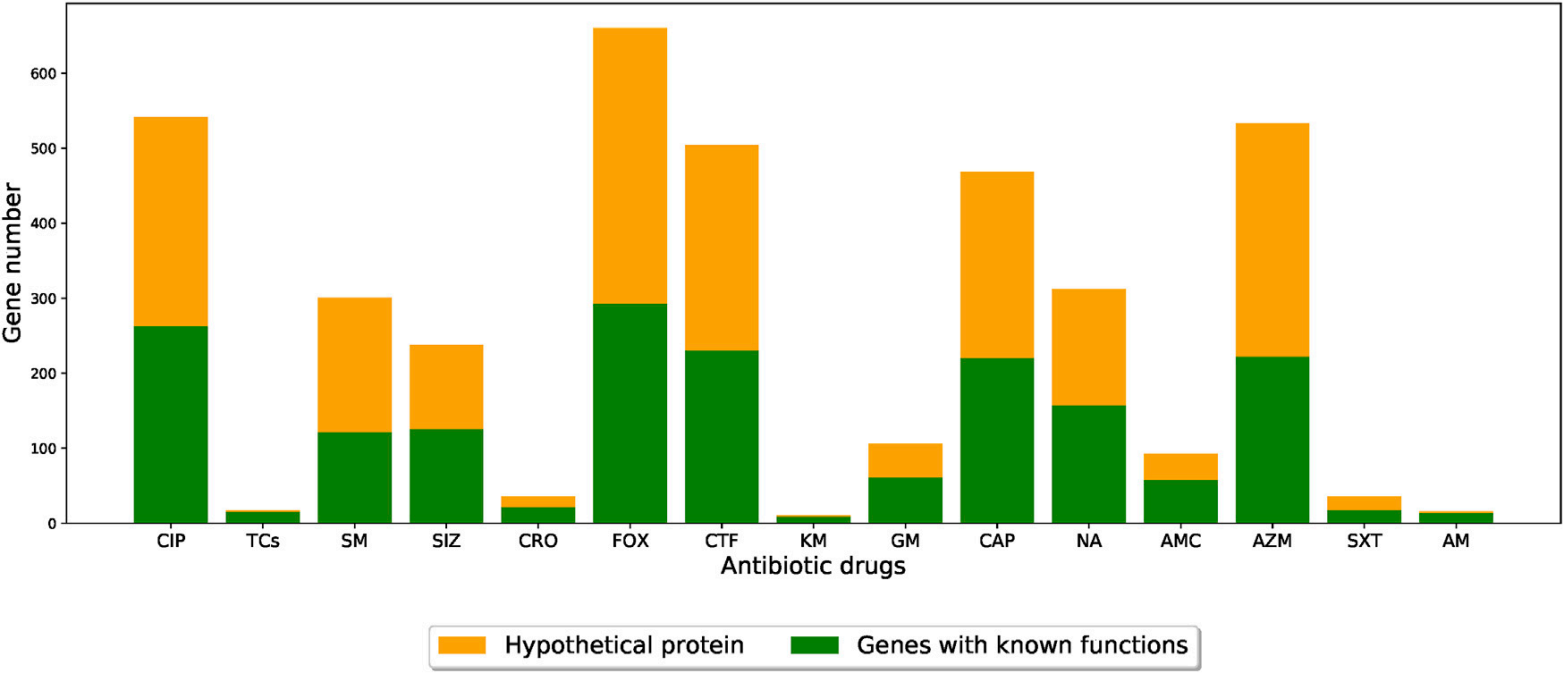
Barplots showing the number of XGBoost-selected genes with known and unknown functional annotations for different drug resistance datasets. The orange parts indicate the proportion of functionally-unknown genes while the green parts represent other functional annotations. (CIP: ciprofloxacin; TCs: tetracycline; SM: streptomycin; SIZ: sulfisoxazole; CRO: ceftriaxone; FOX: cefoxitin; CTF: ceftiofur; KM: kanamycin; GM: gentamicin; CAP: chloramphenicol; NA: nalidixic acid; AMC: amoxicillin/clavulanic acid; AZM: azithromycin; SXT: trimethoprim/sulfamethoxazole; AM: ampicillin).

The functional roles of these genes were determined by gene annotation provided by PATRIC. Among the functions of the genes selected by the feature selection algorithm, the most abundant one was hypothetical proteins (i.e., functionally unknown), as shown in Figure 5 (detailed functional occurrences of the XGBoost-selected genes were provided in Supplementary Table S7). This result suggested that genes with uncharacterized mechanisms may also play important roles in the drug resistances, and that the XGBoost-selected genes, which were good predictors for AMR phenotypes, may serve as potential candidates for uncovering novel AMR functionalities.

**FIGURE 6:**
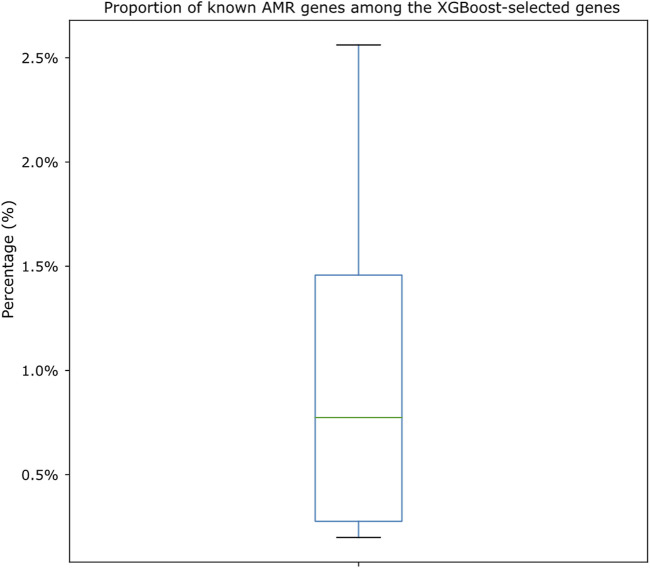
Boxplot showing the proportion of genes shared by both known AMR gene sets and XGBoost-selected gene sets. The proportions are calculated by dividing the number of shared genes against the known AMR gene numbers for each drug dataset. Actual numbers of gene distributions can be found in Supplementary Table S8 and the Venn diagrams shown in Supplementary Figure S4.

We also cross-compared the proportion of XGBoost-selected genes that belonged to known AMR genes (i.e., genes that appeared both in the XGBoost-selected gene set and in the known AMR gene set for each drug). As shown in Figure 6, we identified that, among the known AMR genes, the proportion of genes also identified by the XGBoost algorithm was very low (from as low as 0.20% to as high as 2.56%; Supplementary Table S8). The shared amounts of genes were shown as Venn diagrams for all 15 drugs in Supplementary Figure S4. Annotations of known AMR genes (Supplementary Tables S9, S10) also revealed that certain genes may be more crucial for *S. enterica* drug resistances, in which some are more well-known [such as TetA, TetB, TetC, and TetR for tetracycline resistance (Akiyama et al., 2013)] while others may worth more investigation (for example, a sensor protein identified for ampicillin resistance). A closer look also showed potential cross resistances such as the identification of chloramphenicol/florfenicol resistance MFS efflux pump protein (annotated as FloR family) in ampicillin and trimethoprim/sulfamethoxazole resistance datasets, hinting these genes may contribute to multiple resistance mechanisms.

## Discussion

In this study we attempted to build machine learning models for predicting MIC values for *S. enterica* strains. Our purpose, as was also illustrated in Introduction, was to avoid categorically predicting whether strains were “resistant” or “susceptible” to certain drugs since the breakpoints were determined and may be revised by governing institutes. Once breakpoint revision happens, the prediction of categories would not succeed anymore and the model would have to be retrained. In addition different protocols for microdilution (CLSI, EUCAST, etc.) may influence the interpretation of AMR breakpoints. By contrast, in case of MIC-value prediction, any conclusion on phenotypes (susceptible/resistant) is left to the applying person. In addition, that information is also useful in case that no breakpoints exist, e.g., for assessing MIC-values that differ from the epidemiological cut-off values (ECOFFs) (European Committee for Antimicrobial Susceptibility Testing, 2017). In our results we showed that the MIC values can be predicted with very high accuracy using bacterial pan-genome gene presence/absence patterns, and that selecting relevant features is helpful in conducting MIC regression tasks. Most of the MIC prediction tasks in this study achieved very good performances, in which more than half of the datasets reached *R* ^2^ > 0.9, indicating that the selected features were able to explain more than 90% of the variances. Only three out of 15 dataset underperformed others (*R* ^2^ < 0.5); however the *p*-value yielded from Pearson correlation analysis (Supplementary Table S5, in which *p*-values for all drug datasets were << 0.001) still showed very high correlation between predicted and actual MIC values, supporting the ability of the XGBoost-selected genes in obtaining good regression outcome.

Since MIC values were determined using lab typing methods, different lab typing methods (disk diffusion, broth microdilution, etc.) may create potential biases for building MIC prediction models. We checked the lab typing methods of the strains used in this study and found that the majority of lab typing method was “broth microdilution” that accounted for 86.83% of all MIC records. Meanwhile 13.16% of the records were annotated as “MIC” in their lab typing method field (indicating that their typing method was unknown), and only 0.01% of the entries were annotated as “disk diffusion”. Since the majority of the lab typing method was “broth microdilution,” we therefore concluded that the biases caused by lab typing method should be very small and included all records regardless of their lab typing methods for analysis.

By plotting the side-by-side comparison of the predicted and actual MIC values, as shown in Figure 3, we again observed very good prediction power of the XGBoost-selected genes. Since the plot in Figure 3 aggregated MIC data from 15 drug tables, we plotted the results for separate drugs. As shown in Supplementary Figure S3, most of the drugs achieved satisfactory prediction results. We note that only entries that were integers (both positive and negative ones) after the log2-transformation were included in the boxplots in both Figure 3; Supplementary Figure S3. The reason was to show the clear regression outcome through clean boxplot representation. Certainly there were non-integer log2-transformed MIC; however these entries accounted for only 17.82% (entry numbers for integer and non-integer are 36,558 and 7,925 respectively) for all datasets. We emphasize that even though those non-integer log2-transformed MIC entries were not plotted in Figure 3, the excellent *R* ^2^ and Pearson correlation analysis (Supplementary Tables S3–S5) results showed that overall the XGBoost feature selection-based regression model worked very well on all entries.

Since there were still three drug resistance prediction tasks (ciprofloxacin, azithromycin, and nalidixic acid) that underperformed others (i.e., *R* ^2^ < 0.5) and that these were among the commonly prescribed antibiotics for treating *S. enterica* infection (Khadka et al., 2021), we checked possible reasons including strain numbers, gene numbers, and MIC distributions in order to find some clues. We found that two of the three drug resistance datasets (azithromycin and nalidixic acid) consisted of fewer strains than most other datasets (Supplementary Table S2), indicating that the size of dataset may be one of the reasons. This is however not the case for ciprofloxacin resistances; as a result we checked the MIC distribution for the drug datasets and found that ciprofloxacin had the lowest standard deviation (0.31; see Supplementary Table S11), which might explain the underperformed prediction for this drug since the MIC distribution was centralized only at certain ranges. We note that azithromycin MIC standard deviation was also among the lowest ones among all 15 drugs, which might also partly explain the underperformed MIC prediction of this dataset in addition to having fewer strains.

One may also notice that in Figure 3, the prediction of MIC = 2^8^ entries seems to be not as precise as other entries. We found that the reason was related to sulfisoxazole resistance prediction, in which the results shown in Supplementary Figure S3 suggested that the sulfisoxazole dataset was the only one that consisted of entries with MIC = 2^8^. Meanwhile we also observed that sulfisoxazole dataset had the highest MIC standard deviation (222.53) compared to other datasets, in which the highly deviated MIC distribution may also disturb the regression tasks. We therefore concluded that the MIC distribution may also affect model training and prediction tasks.

The prediction accuracy of the gene sets showed that the XGBoost-selected genes performed better than all genes and known AMR genes, indicating that the XGBoost feature selection method was able to extract relevant protein-coding genes for building better regression model. We note that the improvement was not statistically significant, as the Wilcoxon rank sum test revealed that the *R* ^2^ was not significantly different (*p* = 0.19 and 0.07 for comparing XGBoost-selected genes against all genes and known AMR genes respectively). We however emphasize that the regression performances were mostly improved for the majority of the drugs in terms of *R* ^2^, Pearson correlation analysis, and RMSE (Supplementary Tables S3–S5), indicating that genes identified by feature selection may serve as better predictors compared to known AMR genes.

Since *S. enterica* can be classified into a lot of serovars, we checked whether the MICs of different serovars can also be predicted. We however identified that the strain numbers of most serovars, as shown in Supplementary Table S12, are too few to be subjected to the XGBoost algorithm for training purpose. Even for Typhimurium serovar dataset, in which about a hundred of strains can be identified for each drug resistance, the number was still too few (about only 1/10) compared to the original *S. enterica* datasets. In addition we also found that the host types (i.e., where the *S. enterica* strains were isolated) of most strains were not annotated (Supplementary Table S13). We tried to perform regression on three serovar-specific datasets with higher strain counts (Typhimurium, Kentucky, and Heidelberg) and, not surprisingly, found that the overall regression performances were lower than the original *S. enterica* datasets, as shown in Supplementary Tables S14–S16. We however note that the XGBoost feature selection-based method proposed in this manuscript still reached the highest accuracy compared to all genes and known AMR genes for most of the drug datasets, indicating that the XGBoost-based method was still superior to others. We also checked whether we can extract strains isolated from human; however the strain numbers were too few (also shown in Supplementary Table S12) to be trained for machine learning prediction purposes.

By checking the annotations of the genes extracted by XGBoost algorithm, we found that more than half of the genes were functionally unknown (Figure 5), hinting that there may be uncharacterized AMR mechanisms. Since scientists continue to uncover novel resistance mechanism from a variety of bacterial pathogens [e.g., (Sacha et al., 2008; Fraile-Ribot et al., 2017)], our findings may serve as the starting point for MIC prediction and for more detailed downstream gene analysis. We note that similar discovery were also reported in a previous study (Yang and Wu, 2022), in which about half of the genes highly-associated with AMR phenotypes were hypothetical proteins (e.g., unknown function). Since network-based tools such as EcoliNet (Kim et al., 2015), PseudomonasNet (Hwang et al., 2016), or PangenomeNet (Her et al., 2021) can be used to find functional clues for hypothetical proteins, we believe that building gene networks for *S. enterica* species may be helpful in looking for possible functional roles of the genes.

A cross comparison between known AMR genes (extracted using CARD/RGI and Resfams) and XGBoost-selected genes revealed that there were minimal overlaps between these two gene sets (Figure 6; Supplementary Table S8), indicating that most of the XGBoost-selected genes were not known AMR genes. In other words, the XGBoost-selected genes may act as better predictors for AMR pathogens. At current stage we have not deciphered how or why the XGBoost-selected genes performed better than known AMR genes, as the functions of many XGBoost-selected genes were uncharacterized. We also note that known AMR genes performed slightly worse than not just XGBoost-selected genes but also the all-gene set (statistically insignificant; Wilcoxon rank sum test *p*-value = 0.46 and 0.07 for comparing known AMR genes against all genes and XGBoost-selected genes, respectively).

Although in this work we showed that the pan-genome gene presence/absence pattern may be used for predicting MIC values, and that XGBoost feature selection approach was able to improve the prediction outcomes, there were still limitations that we can further improve in our future work. Firstly the pan-genome construction process did not consider single nucleotide polymorphism (SNP), which may also be important in some resistance mechanisms as SNPs may also contribute to drug resistances. Secondly there may be other factors such as the expression of genes (Suzuki et al., 2014) that may influence antimicrobial resistances. Thirdly we did not attempt to check whether genes were located on chromosomes or plasmids since a large proportion of PATRIC genomes were still fragmented into a number of contigs or scaffolds, and that no information can help us identify whether genes were from genomes or plasmids. Since horizontal gene transfer (HGT) is one of the main routes for transferring AMR genes, the ability to identify plasmids may facilitate better recognition of potential AMR genes. Despite these limitations, we would like to emphasize that in this work we still established a workflow that could be used for highly accurate MIC prediction purpose, and we hope that this workflow could facilitate or strengthen the prediction of MICs in order to understand the AMR potentials of isolated pathogenic strains given their genomic sequences.

## Data Availability

The raw (unprocessed) data was downloaded from the PATRIC website (https://www.bv-brc.org/). The processed pan-genome gene presence/absence data can be accessed at https://doi.org/10.6084/m9.figshare.21913689.v1. The accession number of the strains involved in this study is available in the article/Supplementary Material.

## References

[B1] Akiyama T. Presedo J. Khan A. A. (2013). The tetA gene decreases tigecycline sensitivity of *Salmonella enterica* isolates. Int. J. Antimicrob. Agents 42, 133–140. 10.1016/j.ijantimicag.2013.04.017 23746717

[B2] Akova M. (2016). Epidemiology of antimicrobial resistance in bloodstream infections. Virulence 7, 252–266. 10.1080/21505594.2016.1159366 26984779 PMC4871634

[B3] Alcock B. P. Raphenya A. R. Lau T. T. Y. Tsang K. K. Bouchard M. Edalatmand A. (2019). Card 2020: Antibiotic resistome surveillance with the comprehensive antibiotic resistance database. Nucleic Acids Res. 48, D517–D525. 10.1093/nar/gkz935 PMC7145624 31665441

[B4] Altschul S. F. Madden T. L. Schaffer A. A. Zhang J. Zhang Z. Miller W. (1997). Gapped BLAST and PSI-BLAST: A new generation of protein database search programs. Nucleic Acids Res. 25, 3389–3402. 10.1093/nar/25.17.3389 9254694 PMC146917

[B5] Berman H. F. Riley L. W. (2013). Identification of novel antimicrobial resistance genes from microbiota on retail spinach. BMC Microbiol. 13, 272–277. 10.1186/1471-2180-13-272 24289541 PMC3890574

[B6] Botelho J. Schulenburg H. (2021). The role of integrative and conjugative elements in antibiotic resistance evolution. Trends Microbiol. 29, 8–18. 10.1016/j.tim.2020.05.011 32536522

[B7] Brenner F. W. Villar R. G. Angulo F. J. Tauxe R. Swaminathan B. (2000). *Salmonella* nomenclature. J. Clin. Microbiol. 38, 2465–2467. 10.1128/JCM.38.7.2465-2467.2000 10878026 PMC86943

[B8] Brynildsrud O. Bohlin J. Scheffer L. Eldholm V. (2016). Rapid scoring of genes in microbial pan-genome-wide association studies with Scoary. Genome Biol. 17, 238–239. 10.1186/s13059-016-1108-8 27887642 PMC5124306

[B9] Cai J. Luo J. Wang S. Yang S. (2018). Feature selection in machine learning: A new perspective. Neurocomputing 300, 70–79. 10.1016/j.neucom.2017.11.077

[B10] Chen T. Guestrin C. XGBoost: A scalable tree boosting system. in Proceedings of the 22nd ACM SIGKDD international Conference on knowledge Discovery and data mining: Association for computing machinery (2016). 785–794.

[B11] Costa S. S. Guimaraes L. C. Silva A. Soares S. C. Barauna R. A. (2020). First steps in the analysis of prokaryotic pan-genomes. Bioinform Biol. Insights 14, 1177932220938064. 10.1177/1177932220938064 32843837 PMC7418249

[B12] Demuth J. P. De Bie T. Stajich J. E. Cristianini N. Hahn M. W. (2006). The evolution of mammalian gene families. PLoS One 1, e85. 10.1371/journal.pone.0000085 17183716 PMC1762380

[B13] Eng S.-K. Pusparajah P. Ab Mutalib N.-S. Ser H.-L. Chan K.-G. Lee L.-H. (2015). *Salmonella*: A review on pathogenesis, epidemiology and antibiotic resistance. Front. Life Sci. 8, 284–293. 10.1080/21553769.2015.1051243

[B14] European Committee for Antimicrobial Susceptibility Testing (2017). MIC distributions and epidemiological cut-off value (ECOFF) setting. *EUCAST SOP 10.0* [Online]. Available at: http://www.eucast.org/fileadmin/src/media/PDFs/EUCAST_files/EUCAST_SOPs/EUCAST_SOP_10.0_MIC_distributions_and_epidemiological_cut-off_value__ECOFF__setting_20171117.pdf (Accessed 4 2023, 18).

[B15] Fraile-Ribot P. A. Mulet X. Cabot G. Del Barrio-Tofino E. Juan C. Perez J. L. (2017). *In vivo* emergence of resistance to novel cephalosporin-beta-lactamase inhibitor combinations through the duplication of amino acid D149 from OXA-2 beta-lactamase (OXA-539) in sequence type 235 *Pseudomonas aeruginosa* . Antimicrob. Agents Chemother. 61, e01117–17. 10.1128/AAC.01117-17 28674059 PMC5571340

[B16] Frieri M. Kumar K. Boutin A. (2017). Antibiotic resistance. J. Infect. Public Health 10, 369–378. 10.1016/j.jiph.2016.08.007 27616769

[B17] Gibson M. K. Forsberg K. J. Dantas G. (2015). Improved annotation of antibiotic resistance determinants reveals microbial resistomes cluster by ecology. ISME J. 9, 207–216. 10.1038/ismej.2014.106 25003965 PMC4274418

[B18] Her H.-L. Lin P.-T. Wu Y.-W. (2021). PangenomeNet: A pan-genome-based network reveals functional modules on antimicrobial resistome for *Escherichia coli* strains. BMC Bioinforma. 22, 548. 10.1186/s12859-021-04459-z PMC8579557 34758735

[B19] Her H.-L. Wu Y.-W. (2018). A pan-genome-based machine learning approach for predicting antimicrobial resistance activities of the *Escherichia coli* strains. Bioinformatics 34, i89–i95. 10.1093/bioinformatics/bty276 29949970 PMC6022653

[B20] Hombach M. Bloemberg G. V. Bottger E. C. (2012). Effects of clinical breakpoint changes in CLSI guidelines 2010/2011 and EUCAST guidelines 2011 on antibiotic susceptibility test reporting of Gram-negative bacilli. J. Antimicrob. Chemother. 67, 622–632. 10.1093/jac/dkr524 22167240

[B21] Humphries R. M. Abbott A. N. Hindler J. A. (2019). Understanding and addressing CLSI breakpoint revisions: A primer for clinical laboratories. J. Clin. Microbiol. 57, e00203–e00219. 10.1128/JCM.00203-19 30971460 PMC6535595

[B22] Hwang S. Kim C. Y. Ji S. G. Go J. Kim H. Yang S. (2016). Network-assisted investigation of virulence and antibiotic-resistance systems in *Pseudomonas aeruginosa* . Sci. Rep. 6, 26223. 10.1038/srep26223 27194047 PMC4872156

[B23] Hyun J. C. Kavvas E. S. Monk J. M. Palsson B. O. (2020). Machine learning with random subspace ensembles identifies antimicrobial resistance determinants from pan-genomes of three pathogens. PLoS Comput. Biol. 16, e1007608. 10.1371/journal.pcbi.1007608 32119670 PMC7067475

[B24] Kasuya E. (2019). On the use of R and R-squared in correlation and regression. Ecol. Res. 34, 235–236. 10.1111/1440-1703.1011

[B25] Kavvas E. S. Catoiu E. Mih N. Yurkovich J. T. Seif Y. Dillon N. (2018). Machine learning and structural analysis of *Mycobacterium tuberculosis* pan-genome identifies genetic signatures of antibiotic resistance. Nat. Commun. 9, 4306. 10.1038/s41467-018-06634-y 30333483 PMC6193043

[B26] Khadka P. Thapaliya J. Thapa S. (2021). Susceptibility pattern of *Salmonella enterica* against commonly prescribed antibiotics, to febrile-pediatric cases, in low-income countries. BMC Pediatr. 21, 38. 10.1186/s12887-021-02497-3 33446146 PMC7809854

[B27] Khaledi A. Weimann A. Schniederjans M. Asgari E. Kuo T. H. Oliver A. (2020). Predicting antimicrobial resistance in *Pseudomonas aeruginosa* with machine learning-enabled molecular diagnostics. EMBO Mol. Med. 12, e10264. 10.15252/emmm.201910264 32048461 PMC7059009

[B28] Kim H. Shim J. E. Shin J. Lee I. (2015). EcoliNet: A database of cofunctional gene network for *Escherichia coli* . Database (Oxford) 2015, bav001. 10.1093/database/bav001 25650278 PMC4314589

[B29] Knodler L. A. Elfenbein J. R. (2019). Salmonella enterica . Trends Microbiol. 27, 964–965. 10.1016/j.tim.2019.05.002 31160162

[B30] Lees J. A. Galardini M. Bentley S. D. Weiser J. N. Corander J. (2018). pyseer: a comprehensive tool for microbial pangenome-wide association studies. Bioinformatics 34, 4310–4312. 10.1093/bioinformatics/bty539 30535304 PMC6289128

[B31] Li W. Godzik A. (2006). Cd-Hit: A fast program for clustering and comparing large sets of protein or nucleotide sequences. Bioinformatics 22, 1658–1659. 10.1093/bioinformatics/btl158 16731699

[B32] Li X. Lin J. Hu Y. Zhou J. (2020). Parmap: A pan-genome-based computational framework for predicting antimicrobial resistance. Front. Microbiol. 11, 578795. 10.3389/fmicb.2020.578795 33193203 PMC7642336

[B33] Maguire F. Rehman M. A. Carrillo C. Diarra M. S. Beiko R. G. (2019). Identification of primary antimicrobial resistance drivers in agricultural nontyphoidal *Salmonella enterica* serovars by using machine learning. Msystems 4, e00211–e00219. 10.1128/mSystems.00211-19 31387929 PMC6687941

[B34] Mann C. M. Markham J. L. (1998). A new method for determining the minimum inhibitory concentration of essential oils. J. Appl. Microbiol. 84, 538–544. 10.1046/j.1365-2672.1998.00379.x 9633651

[B35] Mccarthy C. G. P. Fitzpatrick D. A. (2019). Pangloss: A tool for pan-genome analysis of microbial eukaryotes. Genes (Basel) 10, 521. 10.3390/genes10070521 31295964 PMC6678930

[B36] Mcinerney J. O. Mcnally A. O'connell M. J. (2017). Why prokaryotes have pangenomes. Nat. Microbiol. 2, 17040. 10.1038/nmicrobiol.2017.40 28350002

[B37] Medini D. Donati C. Tettelin H. Masignani V. Rappuoli R. (2005). The microbial pan-genome. Curr. Opin. Genet. Dev. 15, 589–594. 10.1016/j.gde.2005.09.006 16185861

[B38] Moradigaravand D. Palm M. Farewell A. Mustonen V. Warringer J. Parts L. (2018). Prediction of antibiotic resistance in *Escherichia coli* from large-scale pan-genome data. PLoS Comput. Biol. 14, e1006258. 10.1371/journal.pcbi.1006258 30550564 PMC6310291

[B39] Nair D. V. T. Venkitanarayanan K. Johny A. K. (2018). Antibiotic-resistant *Salmonella* in the food supply and the potential role of antibiotic alternatives for control. Foods 7, 167. 10.3390/foods7100167 30314348 PMC6210005

[B40] Nguyen M. Brettin T. Long S. W. Musser J. M. Olsen R. J. Olson R. (2018). Developing an *in silico* minimum inhibitory concentration panel test for *Klebsiella pneumoniae* . Sci. Rep. 8, 421. 10.1038/s41598-017-18972-w 29323230 PMC5765115

[B41] Parks D. H. Imelfort M. Skennerton C. T. Hugenholtz P. Tyson G. W. (2015). CheckM: Assessing the quality of microbial genomes recovered from isolates, single cells, and metagenomes. Genome Res. 25, 1043–1055. 10.1101/gr.186072.114 25977477 PMC4484387

[B42] Pataki B. A. Matamoros S. Van Der Putten B. C. L. Remondini D. Giampieri E. Aytan-Aktug D. (2020). Understanding and predicting ciprofloxacin minimum inhibitory concentration in *Escherichia coli* with machine learning. Sci. Rep. 10, 15026. 10.1038/s41598-020-71693-5 32929164 PMC7490380

[B43] Pedregosa F. Varoquaux G. Gramfort A. Michel V. Thirion B. Grisel O. (2011). Scikit-learn: Machine learning in Python. J. Mach. Learn. Res. 12, 2825–2830.

[B44] Peterson E. Kaur P. (2018). Antibiotic resistance mechanisms in bacteria: Relationships between resistance determinants of antibiotic producers, environmental bacteria, and clinical pathogens. Front. Microbiol. 9, 2928. 10.3389/fmicb.2018.02928 30555448 PMC6283892

[B45] Prestinaci F. Pezzotti P. Pantosti A. (2015). Antimicrobial resistance: A global multifaceted phenomenon. Pathog. Glob. Health 109, 309–318. 10.1179/2047773215Y.0000000030 26343252 PMC4768623

[B46] Sacha P. Wieczorek P. Hauschild T. Zorawski M. Olszanska D. Tryniszewska E. (2008). Metallo-beta-lactamases of *Pseudomonas aeruginosa*-a novel mechanism resistance to beta-lactam antibiotics. Folia Histochem Cytobiol. 46, 137–142. 10.2478/v10042-008-0020-9 18519228

[B47] Suzuki S. Horinouchi T. Furusawa C. (2014). Prediction of antibiotic resistance by gene expression profiles. Nat. Commun. 5, 5792. 10.1038/ncomms6792 25517437 PMC4351646

[B48] Tonkin-Hill G. Macalasdair N. Ruis C. Weimann A. Horesh G. Lees J. A. (2020). Producing polished prokaryotic pangenomes with the Panaroo pipeline. Genome Biol. 21, 180. 10.1186/s13059-020-02090-4 32698896 PMC7376924

[B49] Wattam A. R. Davis J. J. Assaf R. Boisvert S. Brettin T. Bun C. (2017). Improvements to PATRIC, the all-bacterial bioinformatics database and analysis resource center. Nucleic Acids Res. 45, D535–D542. 10.1093/nar/gkw1017 27899627 PMC5210524

[B50] Yang M.-R. Wu Y.-W. (2022). Enhancing predictions of antimicrobial resistance of pathogens by expanding the potential resistance gene repertoire using a pan-genome-based feature selection approach. BMC Bioinforma. 23, 131–216. 10.1186/s12859-022-04666-2 PMC9011928 35428201

[B51] Yang M. R. Wu Y. W. (2023). A Cross-Validated Feature Selection (CVFS) approach for extracting the most parsimonious feature sets and discovering potential antimicrobial resistance (AMR) biomarkers. Comput. Struct. Biotechnol. J. 21, 769–779. 10.1016/j.csbj.2022.12.046 36698972 PMC9842539

