# Supplementary material for: Using bacterial pan-genome-based feature selection approach to improve the prediction of minimum inhibitory concentration (MIC)
Source: Front Genet. 2023 May 30;14:1054032. doi: 10.3389/fgene.2023.1054032 (PMC10267731; doi:10.3389/fgene.2023.1054032)
Supplement: Supplementary file 2 [file DataSheet1.PDF]

Supplementary Material

Supplementary materials:

Supplementary Figures S1-S4

Supplementary Tables S2-S16

(Supplementary Table S1, S7, S9, S10, and S13 are provided as Excel files)

Supplementary Figure S1

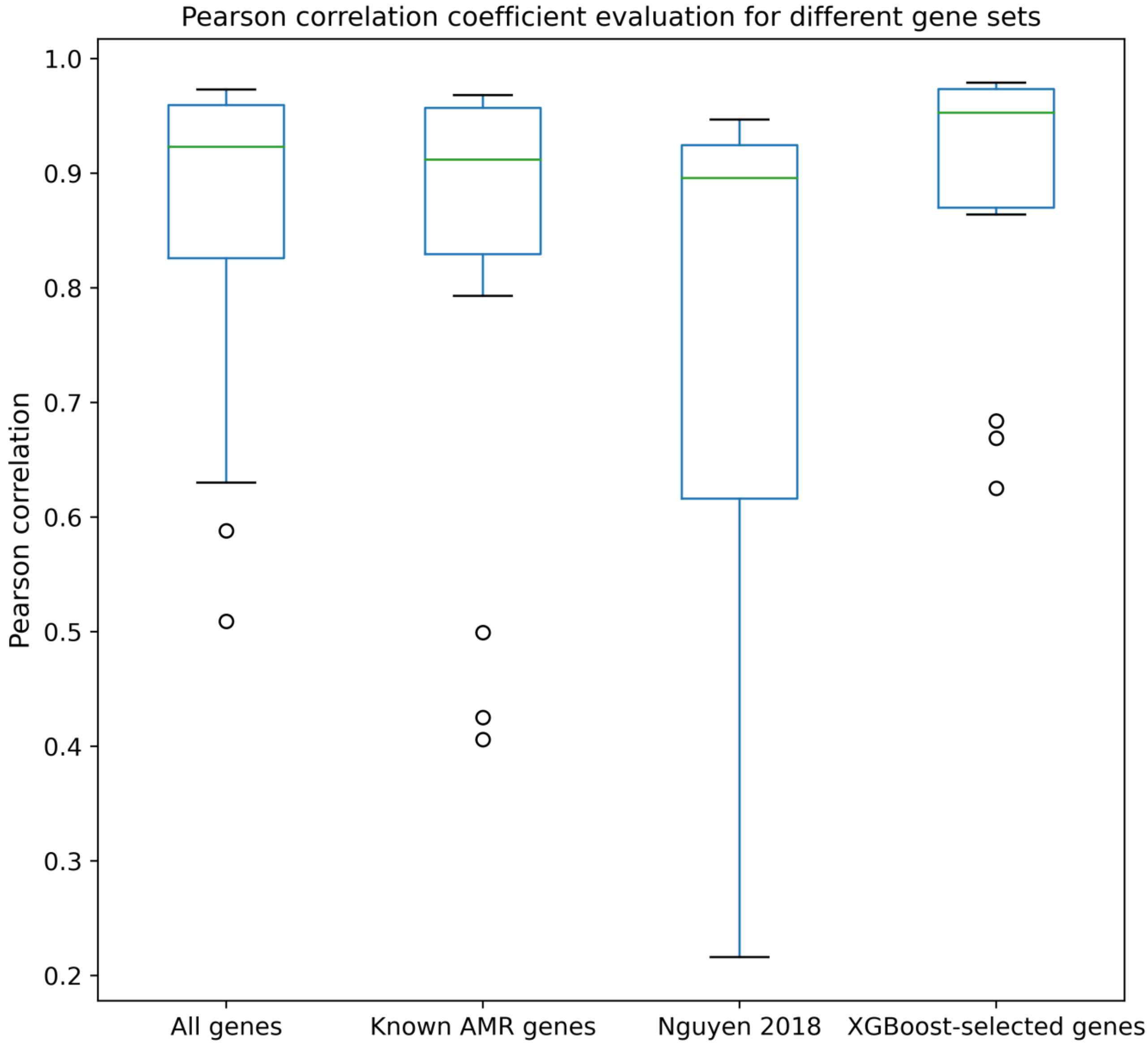

Supplementary Figure S1. Performance evaluations of different gene sets, including all genes, known AMR genes, the k-mer-based approach proposed by (Nguyen 2018), and XGBoost-selected genes. Y-axis indicates Pearson correlation coefficients evaluated by random forest regression through 10-fold cross validation.

Supplementary Figure S2

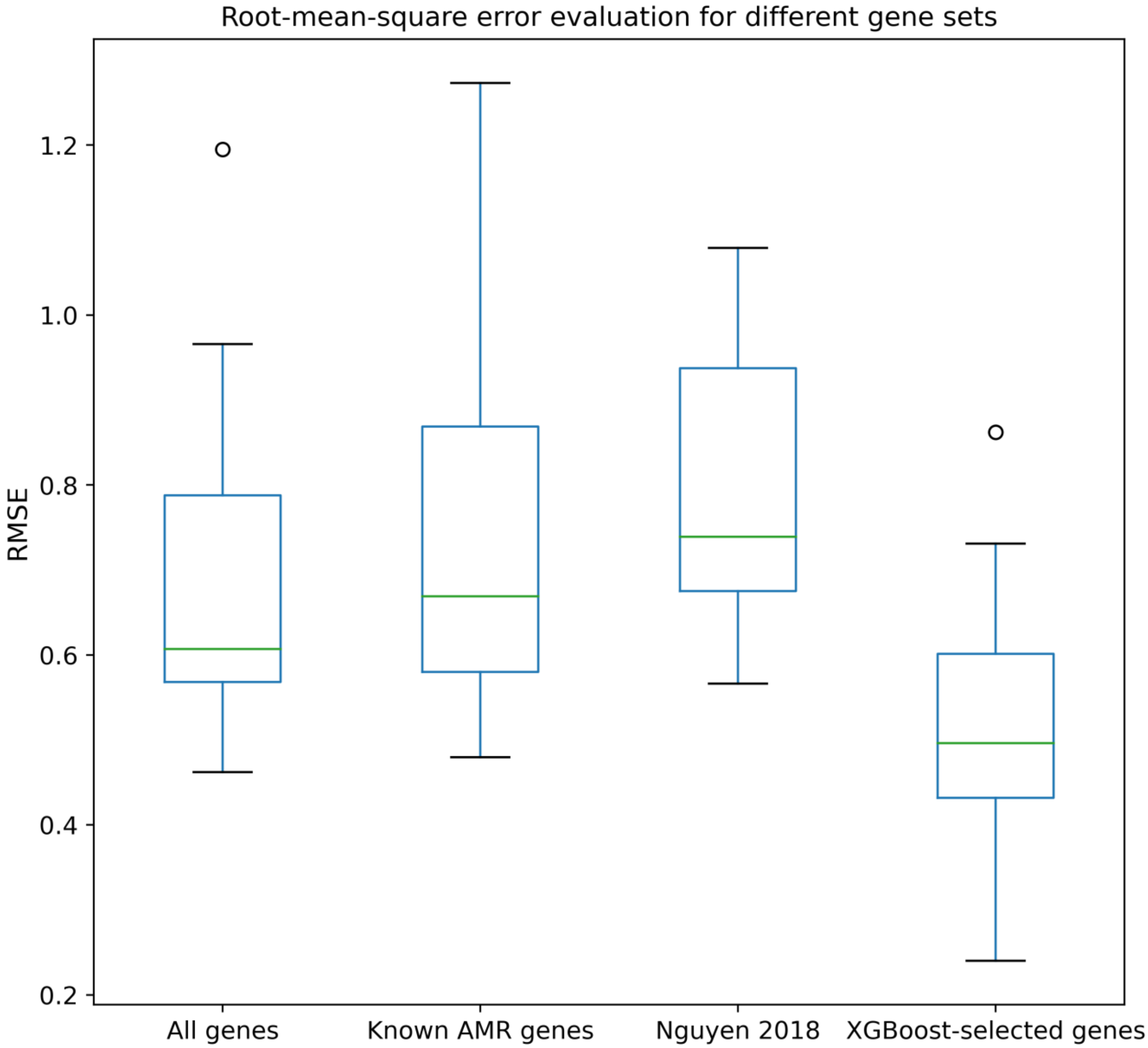

Supplementary Figure S2. Performance evaluations of different gene sets, including all genes, known AMR genes, the k-mer-based approach proposed by (Nguyen 2018), and XGBoost-selected genes. Y-axis indicates RMSE evaluated by random forest regression through 10-fold cross validation.

Supplementary Figure S3

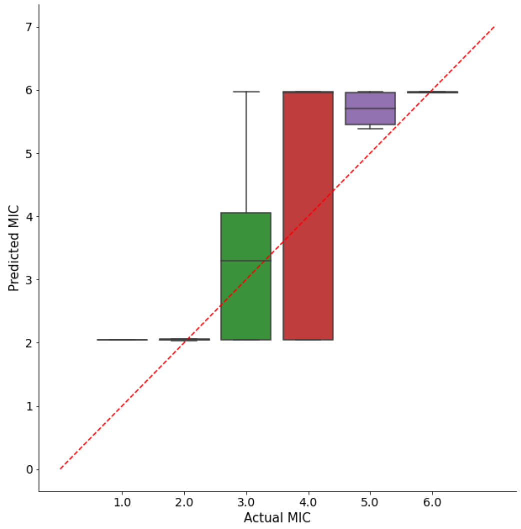

tetracycline

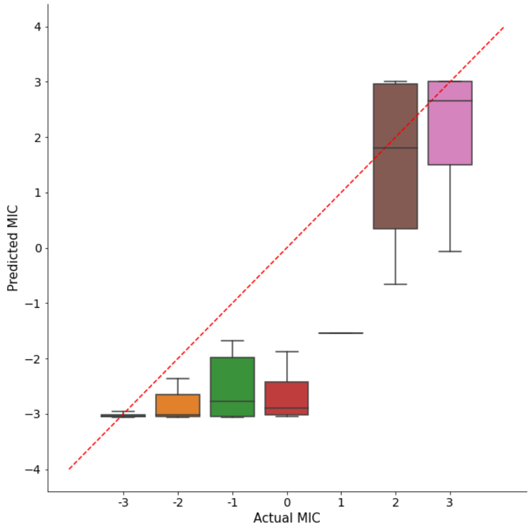

trimethoprim/sulfamethoxazole

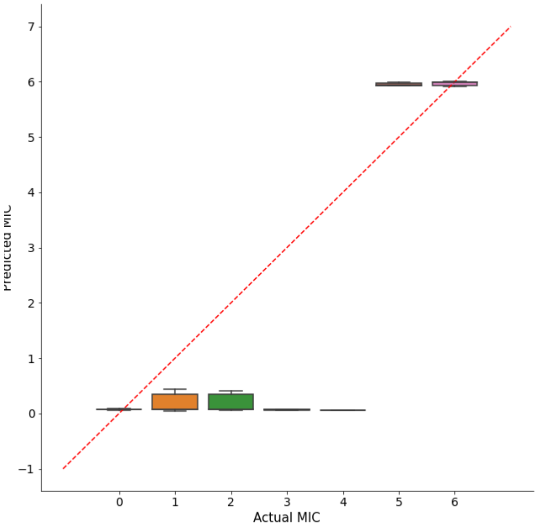

ampicillin

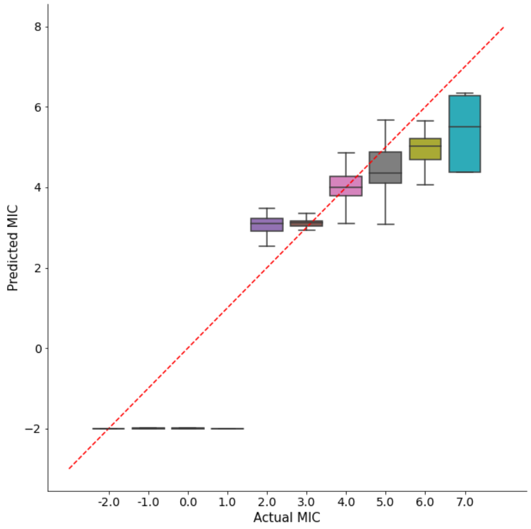

ceftriaxone

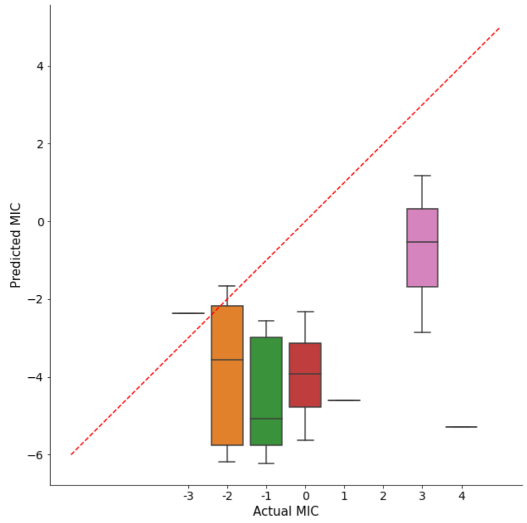

ciprofloxacin

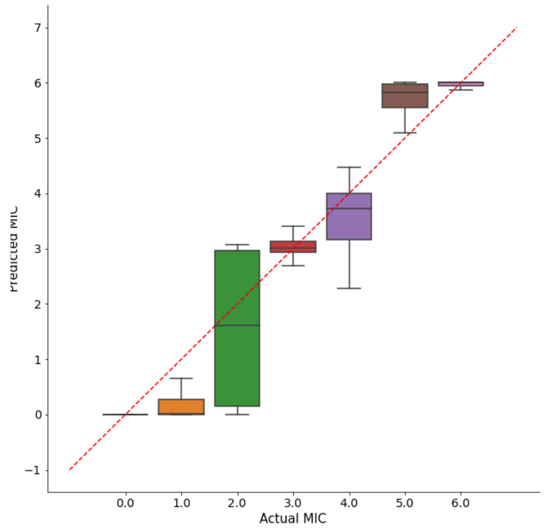

amoxicillin/clavulanic acid

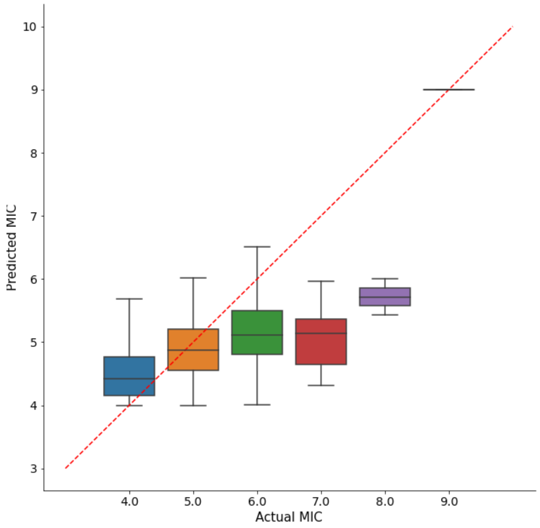

sulfisoxazole

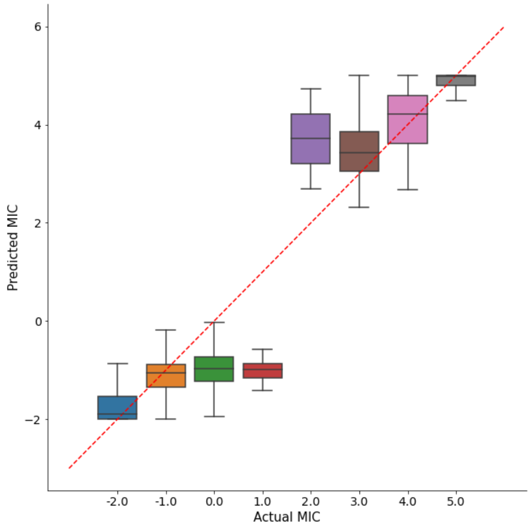

gentamicin

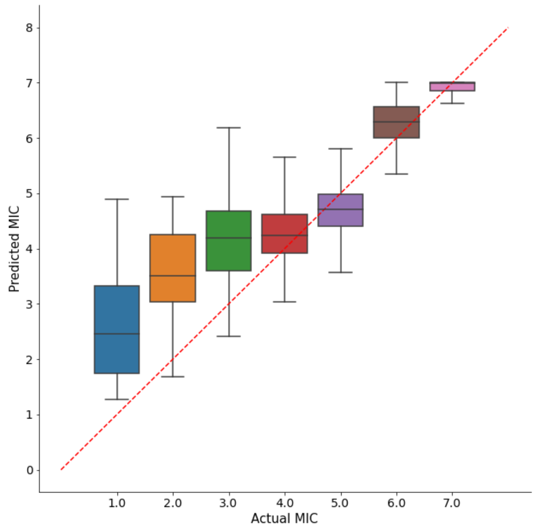

streptomycin

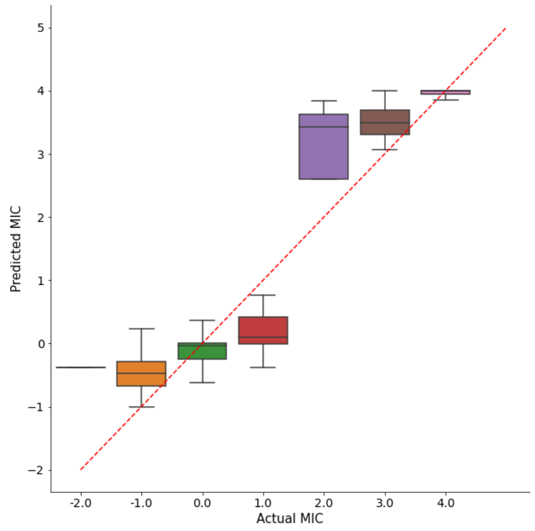

ceftiofur

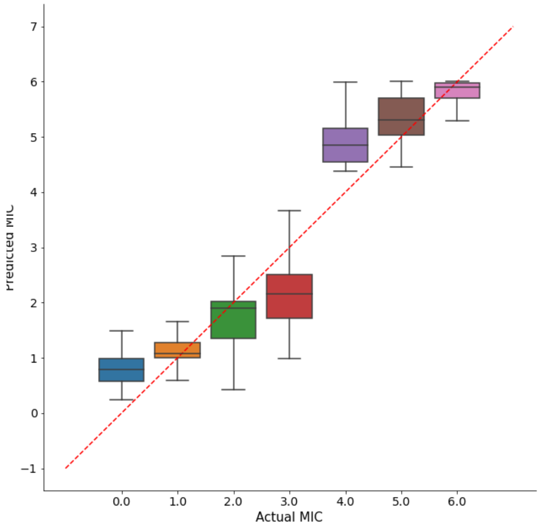

cefoxitin

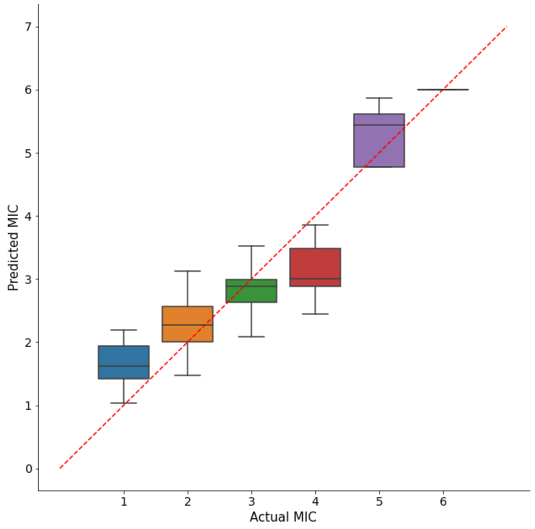

chloramphenicol

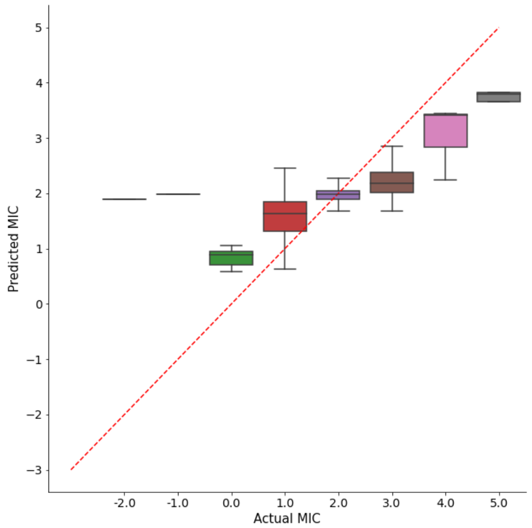

azithromycin

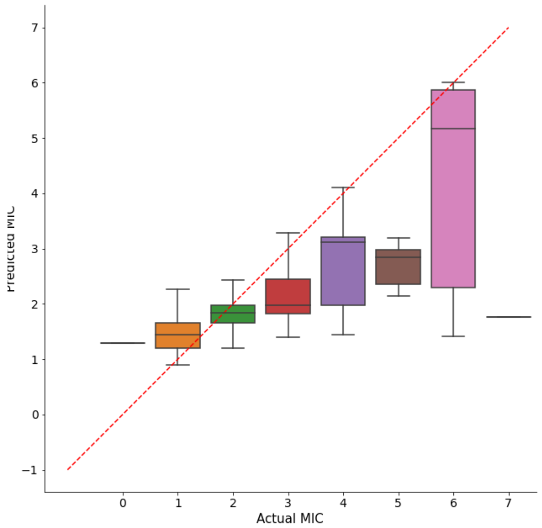

nalidixic acid

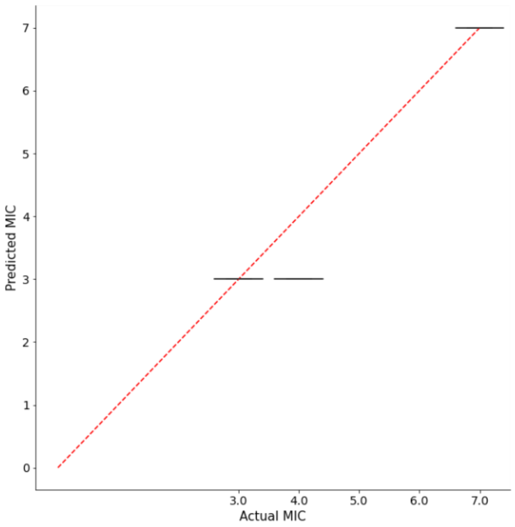

kanamycin

Supplementary Figure S3. The performance evaluations of the 15 individual drugs. Boxplot represent the prediction distribution of a distinct MIC. X-axis is the actual MIC while Y-axis is the predicted MIC for each drug. The drug names are indicated at the bottom of each sub-graph. For better visualization the plot only includes entries with a power of two, in which the exponent is an integer.

Supplementary Figure S4

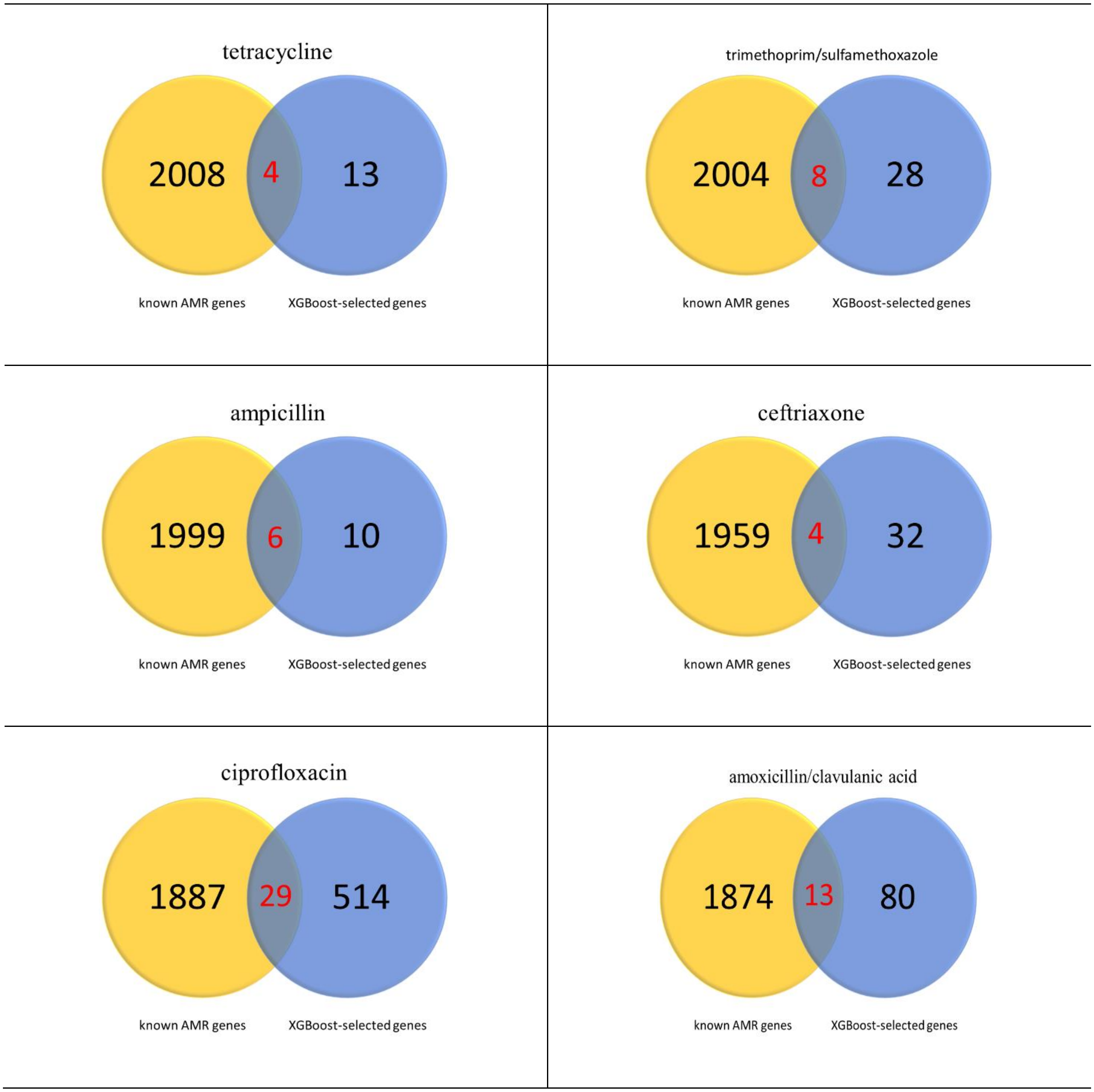

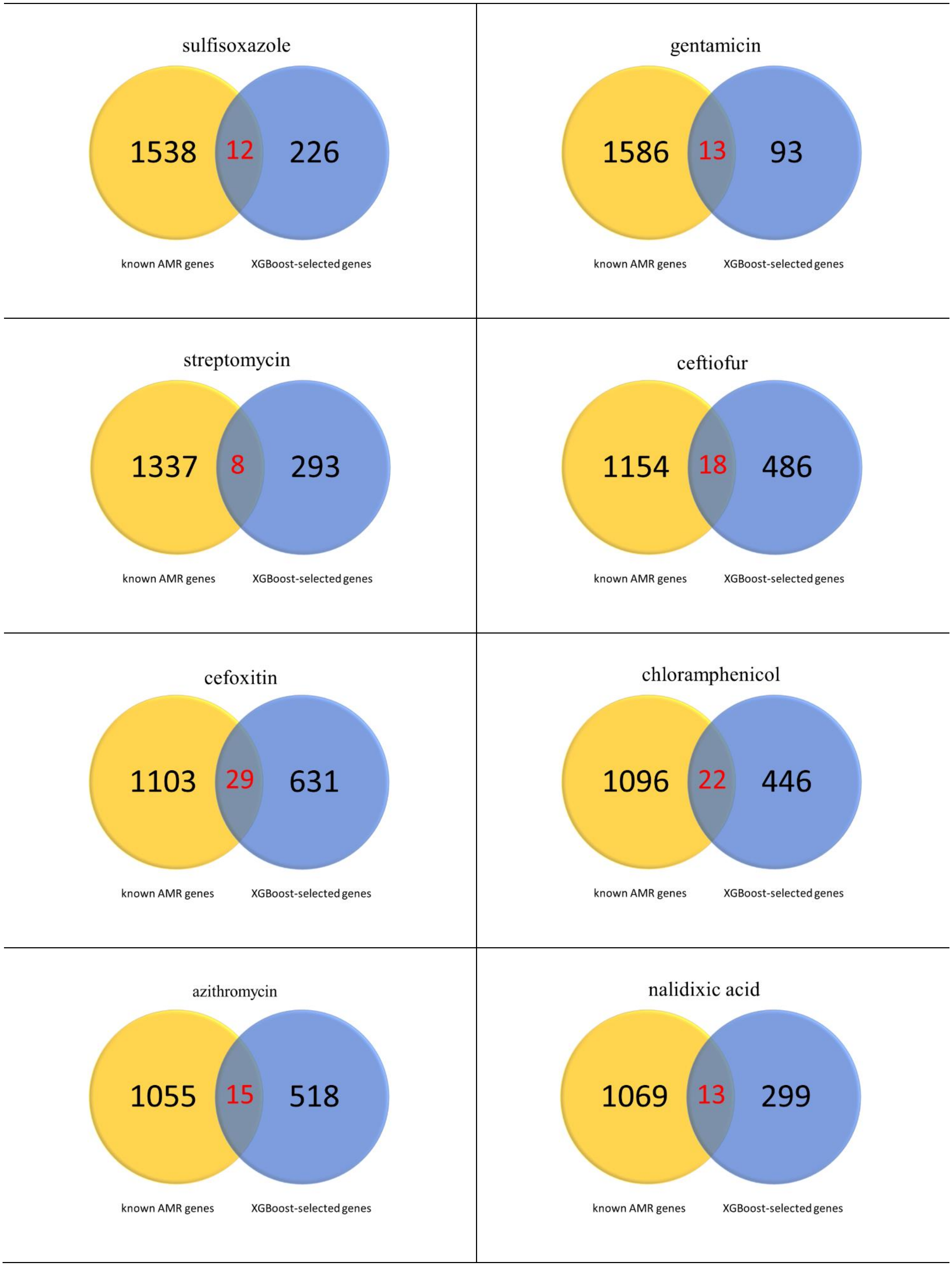
sulfisoxazole
1538
12
226
known AMR genes
XGBoost-selected genes
gentamicin
1586
13
93
known AMR genes
XGBoost-selected genes
streptomycin
1337
8
293
known AMR genes
XGBoost-selected genes
ceftiofur
1154
18
486
known AMR genes
XGBoost-selected genes
cefoxitin
1103
29
631
known AMR genes
XGBoost-selected genes
chloramphenicol
1096
22
446
known AMR genes
XGBoost-selected genes
azithromycin
1055
15
518
known AMR genes
XGBoost-selected genes
nalidixic acid
1069
13
299
known AMR genes
XGBoost-selected genes

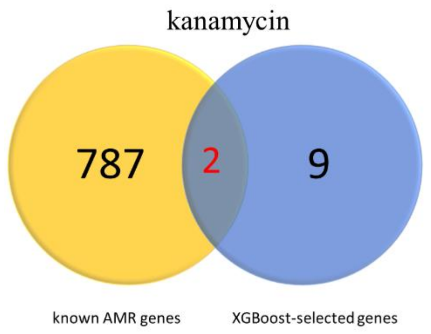

Supplementary Figure S4. Venn diagrams showing the number of genes shared by the known AMR genes and the XGBoost-selected genes for different drugs.

Supplementary Table S2. Number of strains (genomes) with MIC annotation for specific drugs. Yellow entries are drugs that are included in the analysis.

Antibiotic drug	Associated Genome number
tetracycline	4732
trimethoprim/sulfamethoxazole	4641
ampicillin	4515
ceftriaxone	4345
ciprofloxacin	4311
amoxicillin/clavulanic acid	4017
sulfisoxazole	2908
gentamicin	2908
streptomycin	2061
ceftiofur	2040
cefoxitin	1902
chloramphenicol	1754
azithromycin	1654
nalidixic acid	1648
kanamycin	1047
levofloxacin	16
tigecycline	13
ceftazidime	5
ampicillin/sulbactam	3
cefepime	3
imipenem	3
meropenem	3
amikacin	2

aztreonam	2
cefazolin	2
cefotaxime	2
colistin	2
doripenem	2
ertapenem	2
piperacillin/tazobactam	2
polymyxin B	2
tobramycin	2
nitrofurantoin	1

Supplementary Tables S3. Regression performances as evaluated by R^2 for different feature sets of the involved drugs. The feature sets include all genes, known AMR genes, the k-mer-based approach proposed by (Nguyen 2018), and XGBoost-selected genes. Red color indicates the best regression performance among all feature sets.

Drugs	R^2			
	All genes	Known AMR genes	Nguyen 2018[a]	XGBoost-selected genes
tetracycline	0.95	0.93	0.86	0.96
trimethoprim/ sulfamethoxazole	0.58	0.63	0.08	0.76
ampicillin	0.94	0.93	0.88	0.96
ceftriaxone	0.92	0.92	0.80	0.95
ciprofloxacin	0.39	0.22	0.17	0.44
amoxicillin/ clavulanic acid	0.94	0.92	0.89	0.96
sulfisoxazole	0.85	0.83	0.82	0.93
gentamicin	0.81	0.79	0.80	0.88
streptomycin	0.82	0.76	0.58	0.77
ceftiofur	0.92	0.90	0.86	0.94
cefoxitin	0.91	0.89	0.85	0.93
chloramphenicol	0.79	0.74	0.73	0.87
azithromycin	0.26	0.10	0.02	0.39
nalidixic acid	0.34	0.12	0.13	0.47
kanamycin	0.88	0.84	0.69	0.95

[a] The k-mer-based XGBoost regression model proposed by (Nguyen et al. 2018 Scientific Reports).

Supplementary Tables S4. Regression performances as evaluated by root-mean-square error (RMSE) for different feature sets of the involved drugs. The feature sets include all genes, known AMR genes, the k-mer-based approach proposed by (Nguyen 2018), and XGBoost-selected genes. Red color indicates the best regression performance (i.e. lowest RMSE) among all feature sets.

	Root-mean-square error (RMSE)			
Drugs	All genes	Known AMR genes	Nguyen 2018[a]	XGBoost-selected genes
tetracycline	0.58	0.62	0.74	0.42
trimethoprim/ sulfamethoxazole	0.59	0.56	0.80	0.39
ampicillin	0.79	0.87	0.97	0.57
ceftriaxone	0.49	0.48	0.67	0.35
ciprofloxacin	0.78	0.86	0.68	0.56
amoxicillin/ clavulanic acid	0.67	0.74	0.74	0.48
sulfisoxazole	0.97	1.03	0.94	0.63
gentamicin	1.19	1.27	1.08	0.86
streptomycin	0.75	0.86	0.99	0.73
ceftiofur	0.56	0.60	0.73	0.50
cefoxitin	0.61	0.67	0.78	0.56
chloramphenicol	0.60	0.65	0.64	0.46
azithromycin	0.50	0.55	0.57	0.44
nalidixic acid	0.82	0.94	0.94	0.73
kanamycin	0.46	0.50	0.60	0.24

[a] The k-mer-based XGBoost regression model proposed by (Nguyen et al. 2018 Scientific Reports).

Supplementary Tables S5. Regression performances as evaluated by Pearson correlation for different feature sets of the involved drugs. Both correlation coefficients and p-values were estimated. The feature sets include all genes, known AMR genes, the k-mer-based approach proposed by (Nguyen 2018), and XGBoost-selected genes. Red color indicates the best regression performance (i.e. highest correlation coefficent) among all feature sets.

	Pearson correlation coefficient (p-value)			
Drugs	All genes	Known AMR genes	Nguyen 2018[a]	XGBoost-selected genes
tetracycline	0.97 (0.00)	0.97 (0.00)	0.93 (0.00)	0.98 (0.00)
trimethoprim/ sulfamethoxazole	0.76 (0.00)	0.79 (0.00)	0.22 (6.27e^{-50})	0.86 (0.00)
ampicillin	0.97 (0.00)	0.96 (0.00)	0.94 (0.00)	0.98 (0.00)
ceftriaxone	0.96 (0.00)	0.96 (0.00)	0.90 (0.00)	0.97 (0.00)
ciprofloxacin	0.63 (0.00)[b]	0.50 (1.25 e^{-270})	0.47 (3.20e^{-235})	0.67 (0.00)
amoxicillin/ clavulanic acid	0.97 (0.00)	0.96 (0.00)	0.95 (0.00)	0.98 (0.00)
sulfisoxazole	0.92 (0.00)	0.91 (0.00)	0.90 (0.00)	0.95 (0.00)
gentamicin	0.90 (0.00)	0.89 (0.00)	0.90 (0.00)	0.93 (0.00)
streptomycin	0.91 (0.00)	0.87 (0.00)	0.76 (0.00)	0.88 (0.00)
ceftiofur	0.96 (0.00)	0.95 (0.00)	0.93 (0.00)	0.97 (0.00)
cefoxitin	0.95 (0.00)	0.94 (0.00)	0.92 (0.00)	0.96 (0.00)
chloramphenicol	0.89 (0.00)	0.87 (0.00)	0.86 (0.00)	0.93 (0.00)
azithromycin	0.51 (8.40e^{-110})	0.41 (1.23 e^{-66})	0.32 (2.24e^{-40})	0.62 (8.64e^{-180})
nalidixic acid	0.59 (7.90e^{-154})	0.43 (2.25e^{-73})	0.41 (3.38e^{-69})	0.68 (5.70e^{-228})
kanamycin	0.94 (0.00)	0.93 (0.00)	0.84 (4.76e^{-275})	0.97 (0.00)

[a] The k-mer-based XGBoost regression model proposed by (Nguyen et al. 2018 Scientific Reports).

[b] Numbers within the parentheses are p-values for the correlation.

Supplementary Tables S6. The number of gene clusters that were included in the analysis for different feature sets of the involved drugs. The feature sets include all genes, known AMR genes, and XGBoost-selected genes. The k-mer-based approach proposed by (Nguyen 2018) was not included in this analysis since this approach was not based on genes.

	Number of gene clusters		
Drugs	All genes	Known AMR genes	XGBoost-selected genes
tetracycline	68802	2012	17
trimethoprim/ sulfamethoxazole	72168	2012	36
ampicillin	29098	2005	16
ceftriaxone	69476	1963	36
ciprofloxacin	71703	1916	542
amoxicillin/ clavulanic acid	51187	1887	93
sulfisoxazole	71703	1550	238
gentamicin	68861	1599	106
streptomycin	68947	1345	301
ceftiofur	70661	1172	504
cefoxitin	67914	1132	660
chloramphenicol	68922	1118	468
azithromycin	71673	1070	533
nalidixic acid	71813	1082	312
kanamycin	63967	789	11

Supplementary Tables S8. The numbers of known AMR genes and XGBoost-selected genes along with genes appear in both categories (shared genes). The percentages of shared genes indicate the proportion of shared genes among known AMR genes. The k-mer-based approach proposed by (Nguyen 2018) was not included in this analysis since this approach was not based on genes.

Drugs	Known AMR genes	XGBoost-selected genes	Shared genes (known AMR gene proportion)
tetracycline	2012	17	4 (0.20%)
trimethoprim/ sulfamethoxazole	2012	36	8 (0.40%)
ampicillin	2005	16	6 (0.30%)
ceftriaxone	1963	36	4 (0.20%)
ciprofloxacin	1916	542	29 (1.51%)
amoxicillin/ clavulanic acid	1887	93	13 (0.69%)
sulfisoxazole	1550	238	12 (0.77%)
gentamicin	1599	106	13 (0.81%)
streptomycin	1345	301	8 (0.59%)
ceftiofur	1172	504	18 (1.54%)
cefoxitin	1132	660	29 (2.56%)
chloramphenicol	1118	468	22 (1.97%)
azithromycin	1070	533	15 (1.40%)
nalidixic acid	1082	312	13 (1.20%)
kanamycin	789	11	2 (0.25%)

Supplementary Tables S11. The mean and standard deviation of the MIC distribution for different drugs of *Salmonella enterica* resistances.

Drugs	MIC mean	MIC standard deviation
tetracycline	36.03	29.43
trimethoprim/ sulfamethoxazole	0.27	1.03
ampicillin	20.54	28.97
ceftriaxone	3.32	9.79
ciprofloxacin	0.04	0.31
amoxicillin/ clavulanic acid	11.22	20.73
sulfisoxazole	199.57	222.53
gentamicin	3..89	9.28
streptomycin	62.29	47.10
ceftiofur	2.87	4.90
cefoxitin	9.18	16.64
chloramphenicol	9.05	11.87
azithromycin	4.08	2.23
nalidixic acid	4.49	7.23
kanamycin	17.58	32.15

Supplementary Table S12. Number of serovar-specific *Salmonella enterica* strains with MIC annotation for the drugs. Numbers within parentheses indicate number of strains that were isolated from human.

Antibiotic drug	Typhimurium Associated Genome number	Kentucky Associated Genome number	Heidelberg Associated Genome number	Infantis Associated Genome number	Anatum Associated Genome number	Newport Associated Genome number
tetracycline	118 (0)[1]	69 (0)	67 (0)	17 (0)	6 (0)	4 (0)
trimethoprim/ sulfamethoxazole	118 (2)	69 (0)	67 (0)	17 (0)	6 (0)	4 (0)
ampicillin	118 (2)	69 (0)	67 (0)	17 (0)	6 (0)	4 (0)
ceftriaxone	118 (2)	69 (0)	67 (0)	17 (0)	6 (0)	4 (0)
ciprofloxacin	118 (2)	69 (0)	67 (0)	17 (0)	6 (0)	4 (0)
amoxicillin/ clavulanic acid	118 (0)	69 (0)	67 (0)	17 (0)	6 (0)	4 (0)
sulfisoxazole	117 (0)	69 (0)	67 (0)	17 (0)	6 (0)	4 (0)
gentamicin	118 (0)	69 (0)	67 (0)	17 (0)	6 (0)	4 (0)
streptomycin	117 (0)	69 (0)	67 (0)	17 (0)	6 (0)	4 (0)
ceftiofur	117 (0)	69 (0)	67 (0)	17 (0)	6 (0)	4 (0)
cefoxitin	118 (0)	69 (0)	67 (0)	17 (0)	6 (0)	4 (0)
chloramphenicol	117 (1)	69 (0)	67 (0)	17 (0)	6 (0)	4 (0)
azithromycin	117 (0)	69 (0)	67 (0)	17 (0)	6 (0)	4 (0)
nalidixic acid	117 (2)	69 (0)	67 (0)	17 (0)	6 (0)	4 (0)
kanamycin[2]	1 (0)	0 (0)	4 (0)	0 (0)	0 (0)	0 (0)

[1] The first number indicates the number of *S. enterica* strains that was isolated from all sources, and the second number (number within parentheses) is the number of *S. enterica* strains isolated from human (*Homo sapiens*).

[2] Kanamycin resistance was not included in the ongoing serovar-specific analysis (as reported in Supplementary Table S9) due to too few numbers of entries for all serovar datasets.

Supplementary Tables S14. The regression performances (R^2) evaluated for strains annotated as Typhimurium serovar. The feature sets include all genes, known AMR genes, and XGBoost-selected genes. Red colors indicate the best regression performance for the Typhimuium serovar drug dataset.

Drugs	R^2		
	All genes	Known AMR genes	XGBoost-selected genes
tetracycline	0.78	0.90	0.84
trimethoprim/ sulfamethoxazole	0.00	0.00	0.03
ampicillin	0.96	0.98	1.00
ceftriaxone	0.95	0.95	0.96
ciprofloxacin	0.07	0.00	0.12
amoxicillin/ clavulanic acid	0.97	0.98	0.99
sulfisoxazole	0.72	0.97	0.89
gentamicin	0.53	0.64	0.68
streptomycin	0.58	0.42	0.67
ceftiofur	0.91	0.51	0.92
cefoxitin	0.91	0.90	0.92
chloramphenicol	0.53	0.52	0.78
azithromycin	0.00	0.00	0.09
nalidixic acid	0.06	0.00	0.30

Supplementary Tables S15. The regression performances (R^2) evaluated for strains annotated as Kentucky serovar. The feature sets include all genes, known AMR genes, and XGBoost-selected genes. Red colors indicate the best regression performance for the Kentucky serovar drug dataset.

	R^2		
Drugs	All genes	Known AMR genes	XGBoost-selected genes
tetracycline	0.90	0.94	0.93
trimethoprim/ sulfamethoxazole	0.00	0.00	0.00
ampicillin	0.77	0.85	0.93
ceftriaxone	0.96	0.93	0.99
ciprofloxacin	0.00	0.00	0.00
amoxicillin/ clavulanic acid	0.92	0.94	0.96
sulfisoxazole	0.51	0.56	0.73
gentamicin	0.43	0.44	0.69
streptomycin	0.40	0.52	0.48
ceftiofur	0.94	0.94	0.97
cefoxitin	0.86	0.85	0.90
chloramphenicol	0.12	0.01	0.29
azithromycin	0.36	0.50	0.40
nalidixic acid	0.21	0.11	0.45

Supplementary Tables S16. The regression performances (R^2) evaluated for strains annotated as Heidelberg serovar. The feature sets include all genes, known AMR genes, and XGBoost-selected genes. Red colors indicate the best regression performance for the Heidelberg serovar drug dataset.

Drugs	R^2		
	All genes	Known AMR genes	XGBoost-selected genes
tetracycline	0.84	0.98	0.80
trimethoprim/ sulfamethoxazole	0.00	0.00	0.00
ampicillin	0.89	1.00	0.97
ceftriaxone	0.86	0.98	1.00
ciprofloxacin	0.00	0.00	0.00
amoxicillin/ clavulanic acid	0.77	0.97	0.97
sulfisoxazole	0.68	0.69	0.78
gentamicin	0.99	0.94	0.99
streptomycin	0.65	0.67	0.73
ceftiofur	0.80	0.88	0.90
cefoxitin	0.77	0.85	0.87
chloramphenicol	0.04	0.09	0.36
azithromycin	0.00	0.00	0.07
nalidixic acid	0.11	0.00	0.44
